# Supplementary material for: Integrative genomic and transcriptomic analysis for pinpointing recurrent alterations of plant homeodomain genes and their clinical significance in breast cancer
Source: Oncotarget. 2016 Dec 31;8(8):13099–115. doi: 10.18632/oncotarget.14402 (PMC5355080; doi:10.18632/oncotarget.14402)
Supplement: Supplementary file 1 [file oncotarget-08-13099-s001.pdf]

Integrative genomic and transcriptomic analysis for pinpointing recurrent alterations of plant homeodomain genes and their clinical significance in breast cancer

SUPPLEMENTARY FIGURES AND TABLES

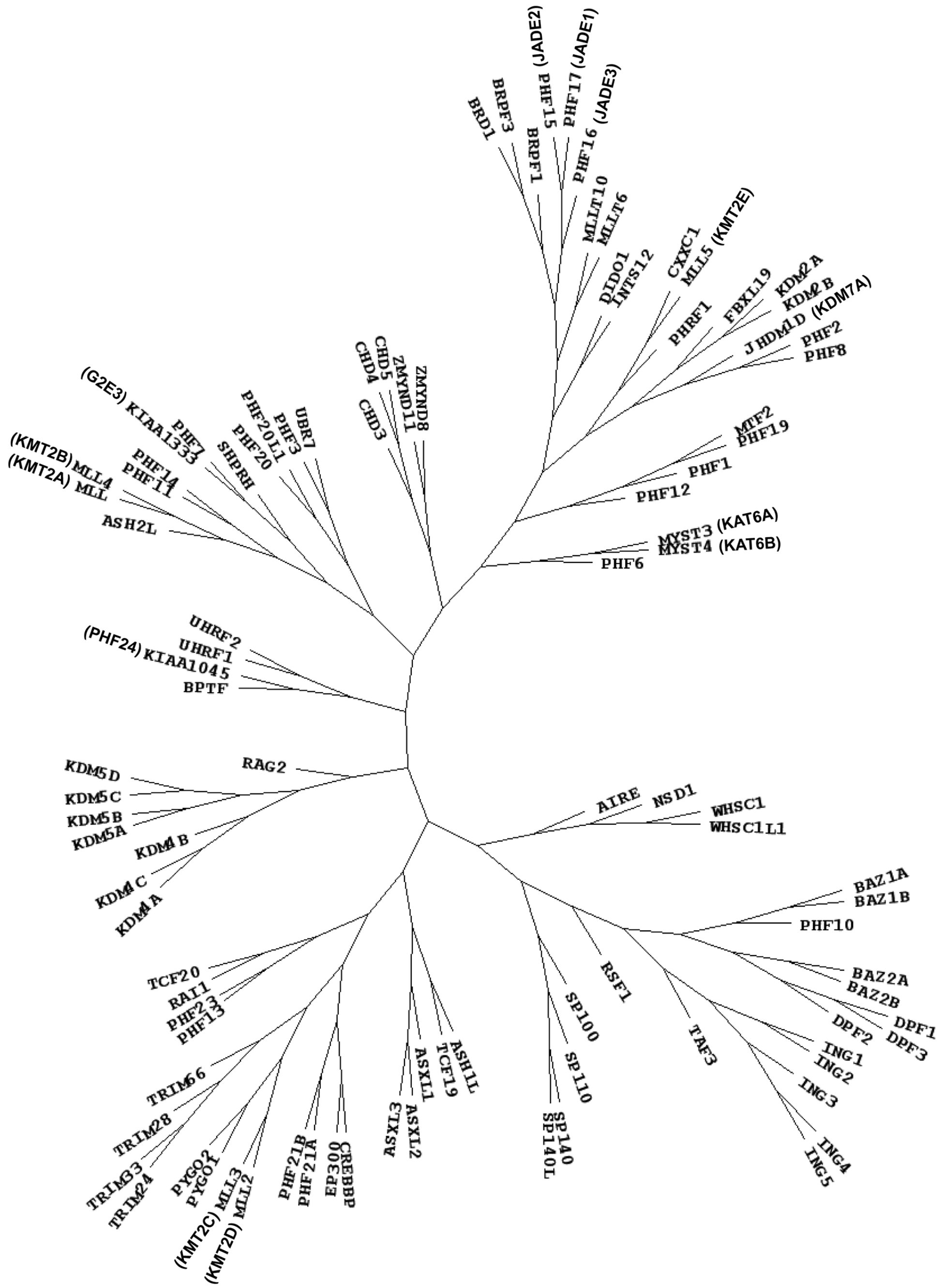

Supplementary Figure 1: Phylogenetic analysis of PHD finger-containing proteins. This image was obtained from ChromoHub (http://www.thesgc.org), a data hub for navigators of chromatin-mediated signaling. The phylogeny outlined in the tree is derived from a CLUSTAL-W multiple sequence alignment of the full-length sequence of the default SWISS-PROT variant.

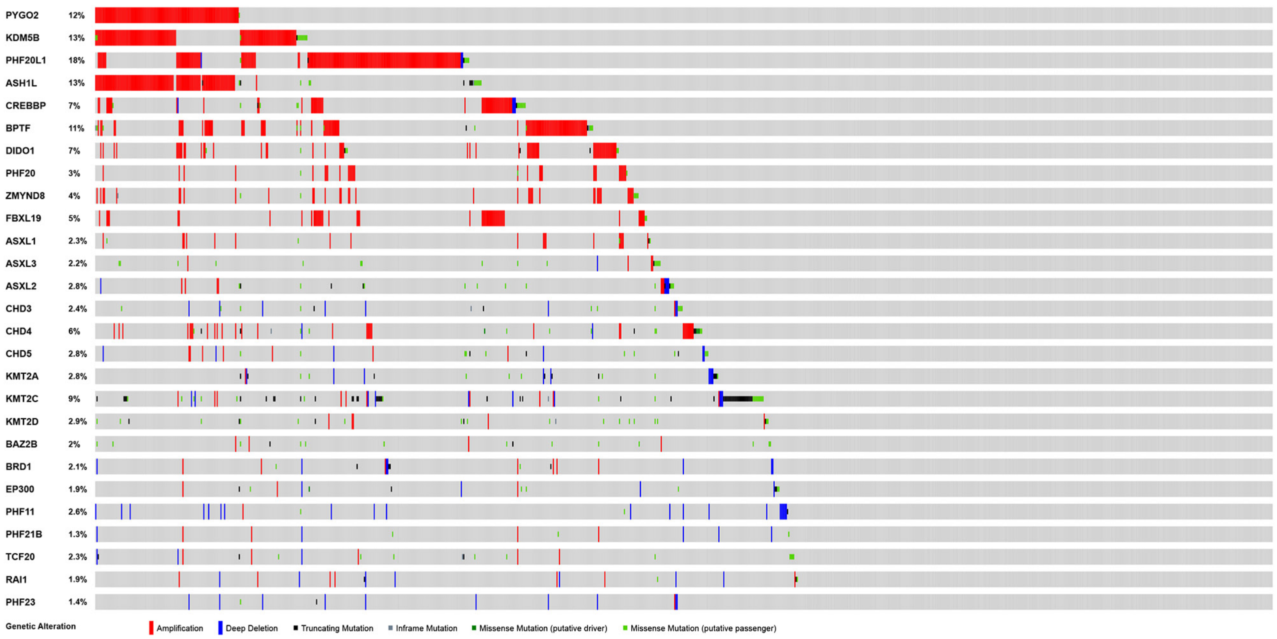

Supplementary Figure 2: High-level amplification, homozygous deletion, and mutation of 27 PHF genes (Table 1) in TCGA breast cancer dataset (n=960). Data are displayed with the Oncoprint tool from cBioPortal.

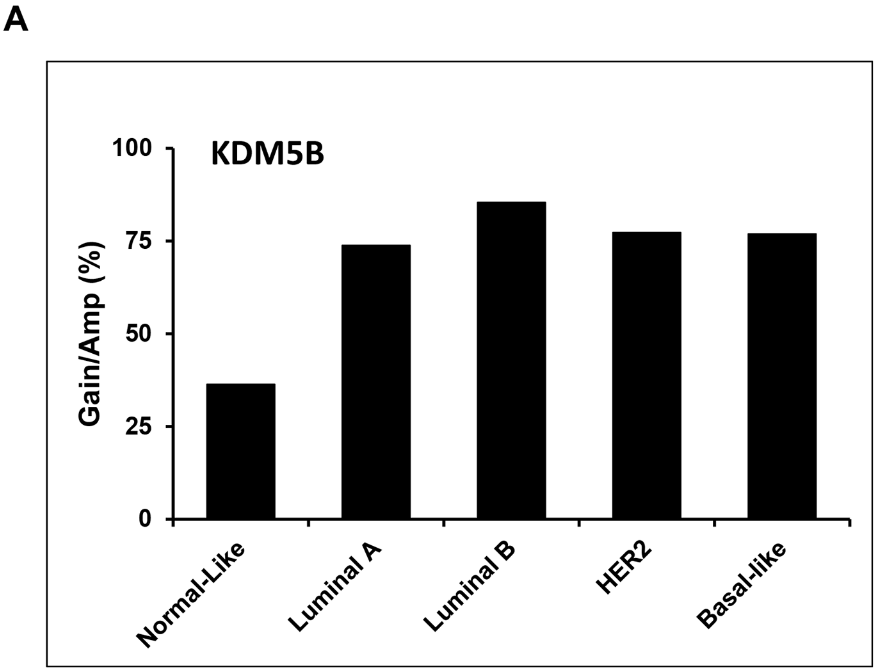

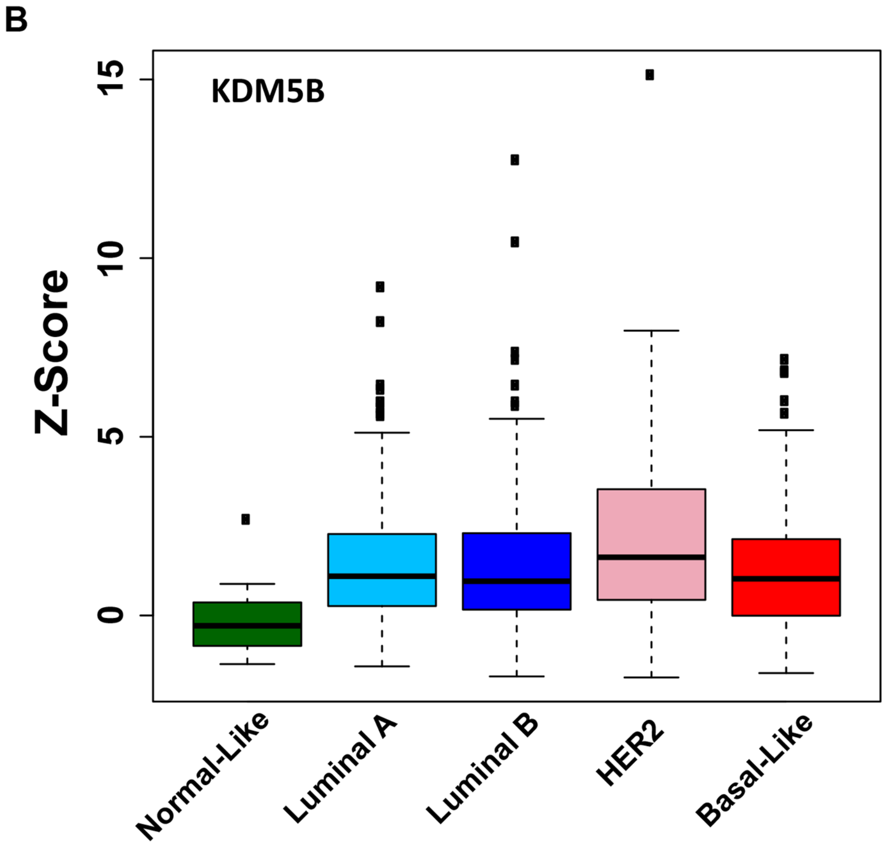

Supplementary Figure 3: A. Frequency of copy number increase and **B.** expression levels of KDM5B across five subtypes of TCGA breast cancer samples.

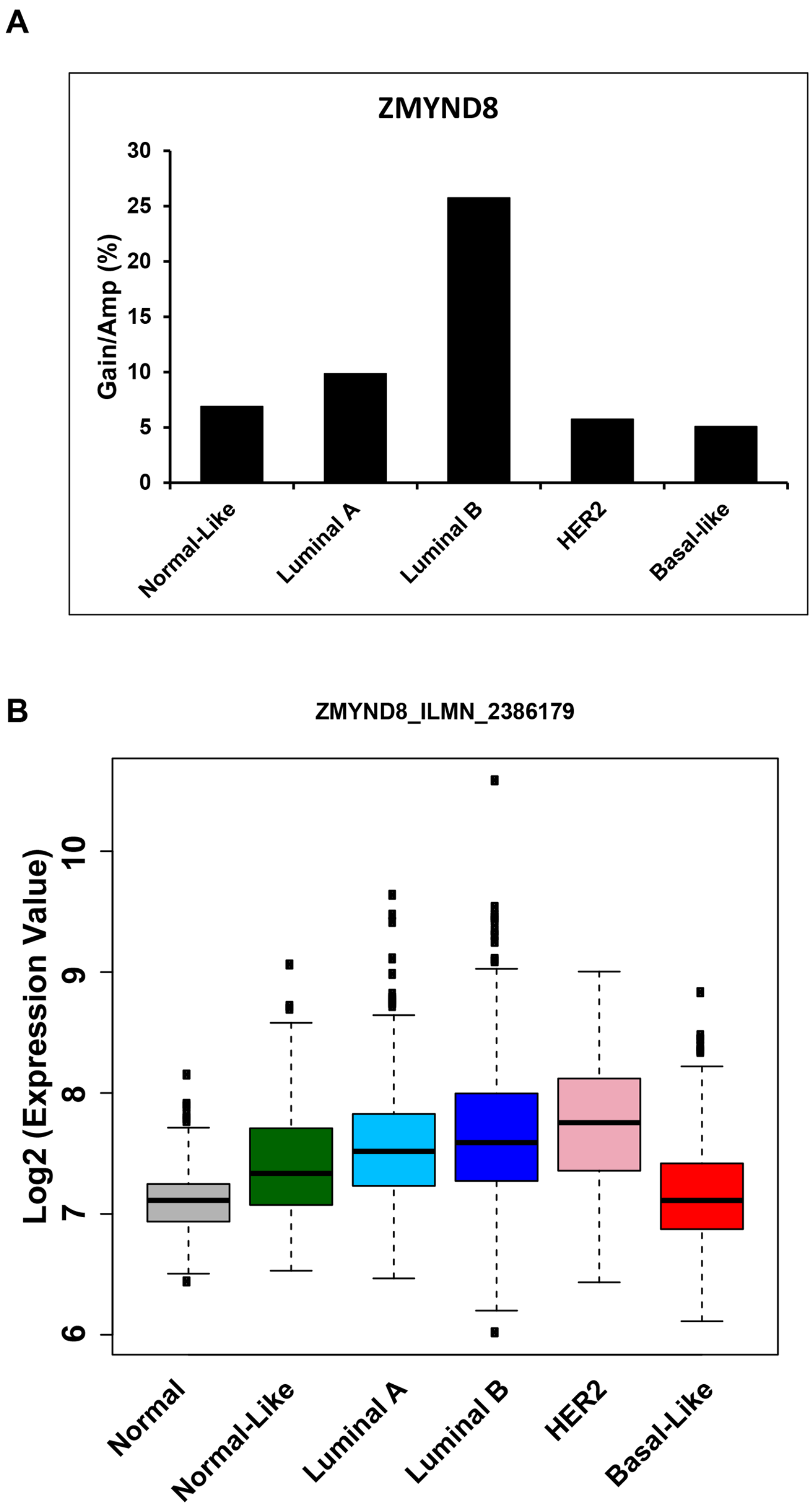

Supplementary Figure 4: A. Frequency of copy number increase and **B.** expression levels of ZMYND8 across five subtypes of METABRIC breast cancer samples. Among the METABRIC breast cancer samples, 1975 had available PAM50 subtype data, including 200 normal-like, 719 Luminal A, 490 Luminal B, 238 HER2+, and 328 basal-like breast cancers. For mRNA expression of ZMYND8, the METABRIC normal breast expression dataset (n= 144) was also used as a non-cancer tissue control.

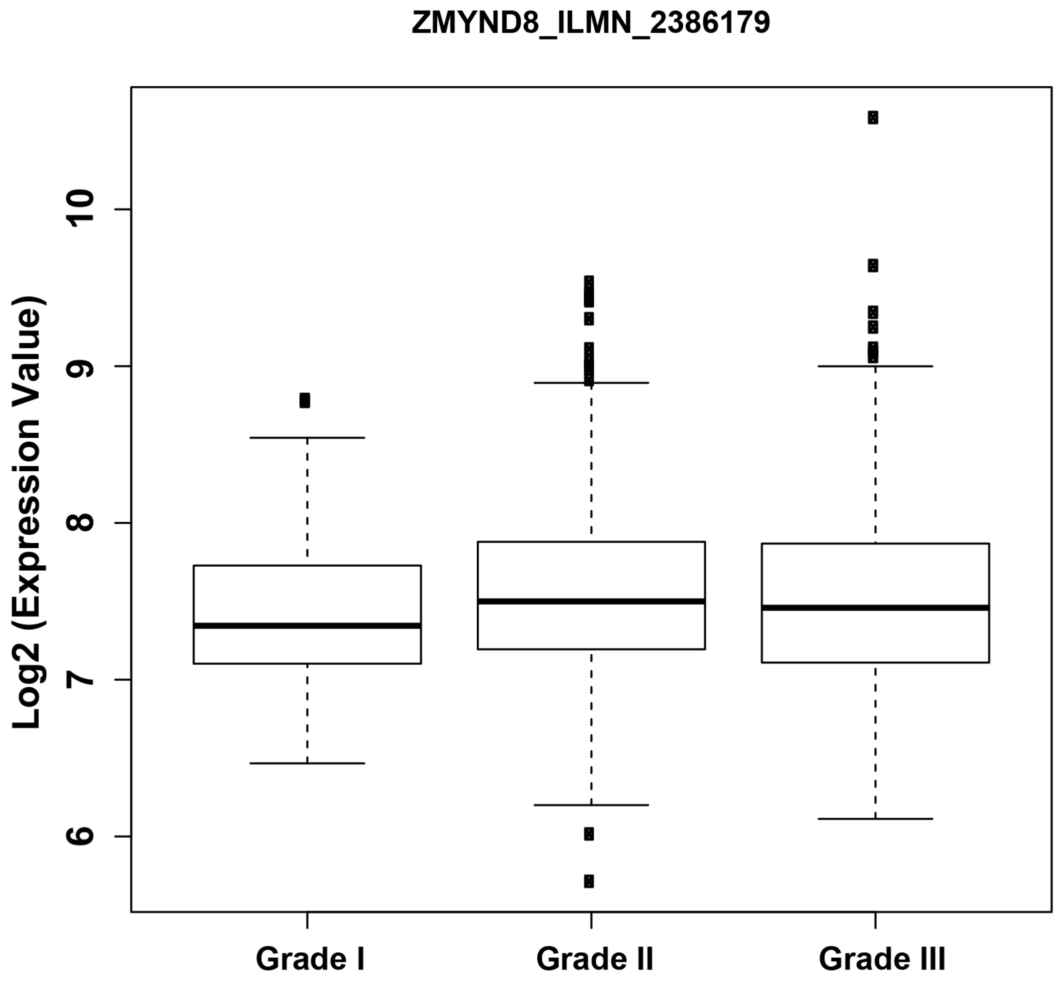

Supplementary Figure 5: Expression levels of ZMYND8 in different grades in METABRIC breast cancer samples.

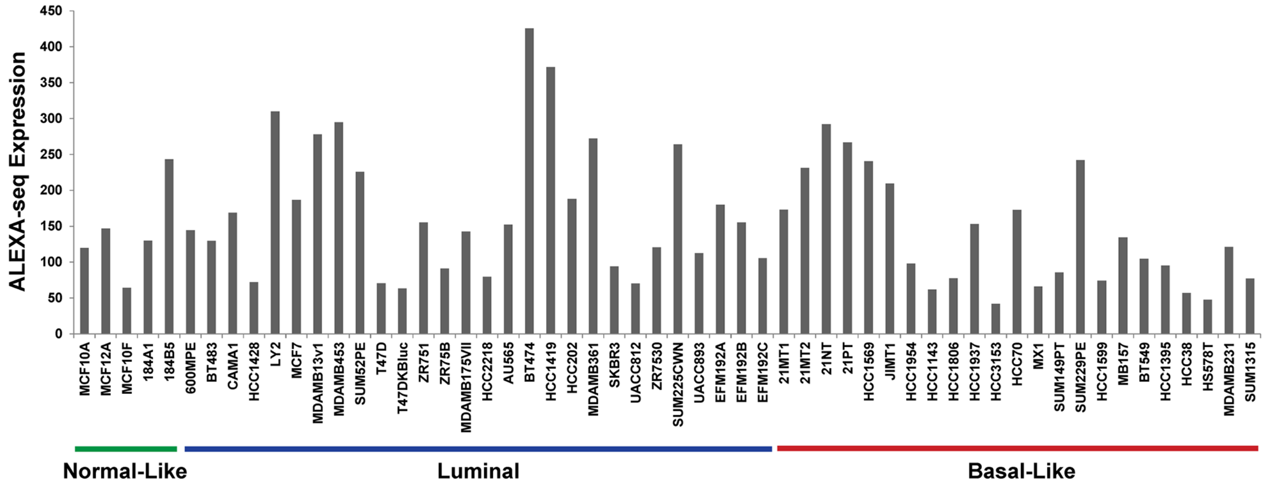

Supplementary Figure 6: mRNA expression levels from RNA-Seq (GSE48216) of ZMYND8 in a panel of 56 breast cancer cell lines compared with 5 normal mammary epithelial cell lines. Based on the transcriptional profiling, cell lines are broadly divided into the following three subgroups: normal mammary epithelial cell lines, Luminal breast cancer cell lines, and basal-like breast cancer cell lines.

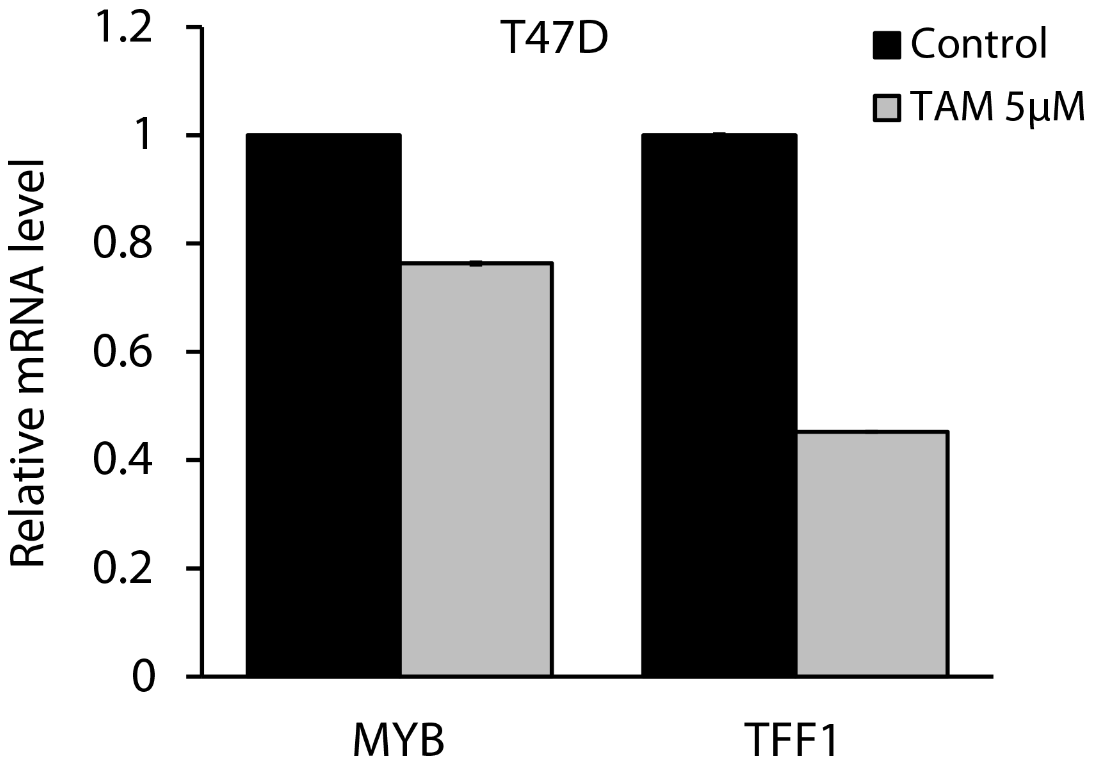

Supplementary Figure 7: qRT-PCR analysis of MYB and TFF1 expression after treatment with tamoxifen (TAM) in T47D cells.

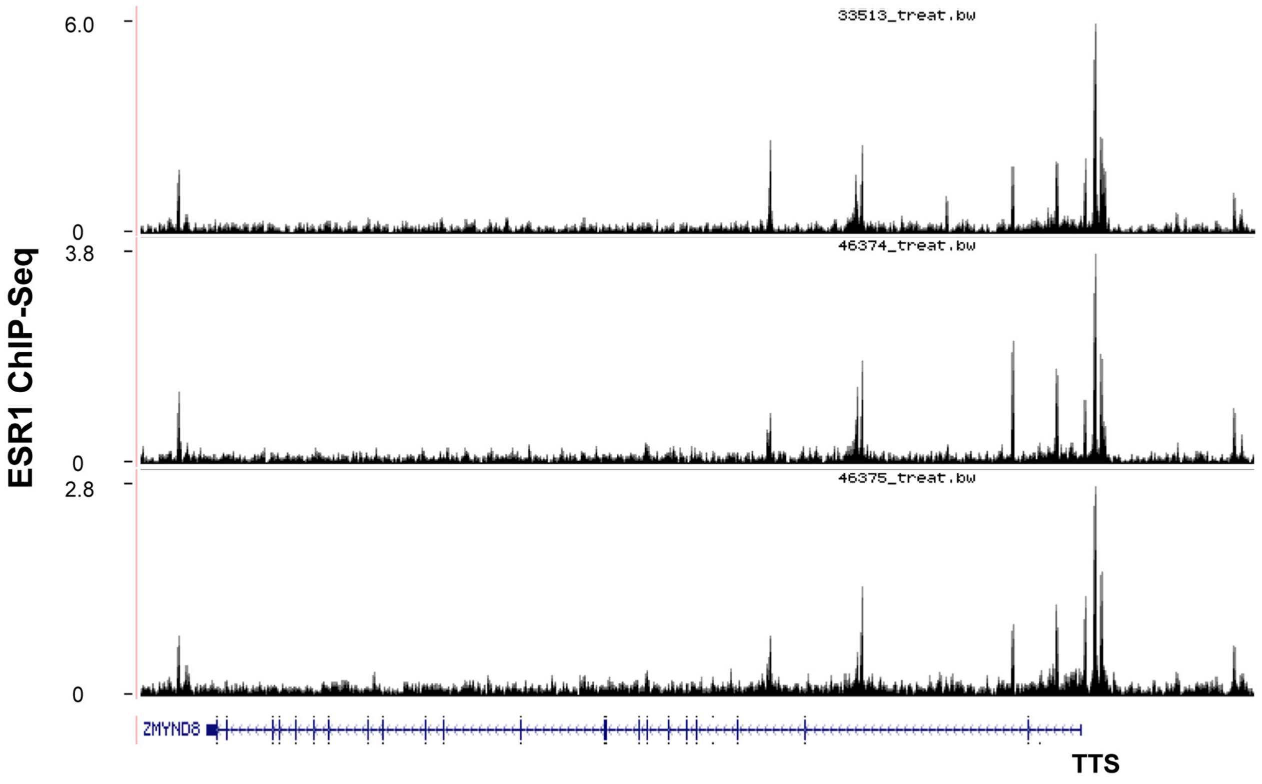

Supplementary Figure 8: ChIP-Seq binding peaks for ESR1 at the ZMYND8 genomic loci in T47D breast cancer cells. The data were obtained from the Cistrome Data Browser (http://dc2.cistrome.org/), and three ChIP-seq data were shown. TSS: Transcription start site.

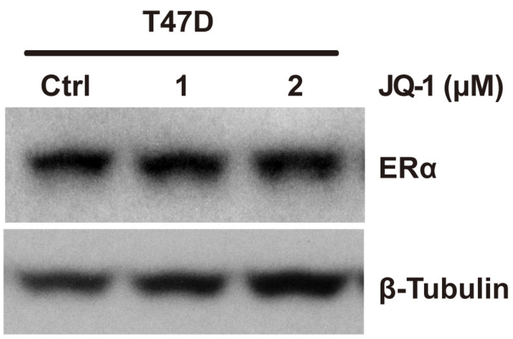

Supplementary Figure 9: Protein level of ERα was measured by western blot after JQ-1 treatment in T47D cells.

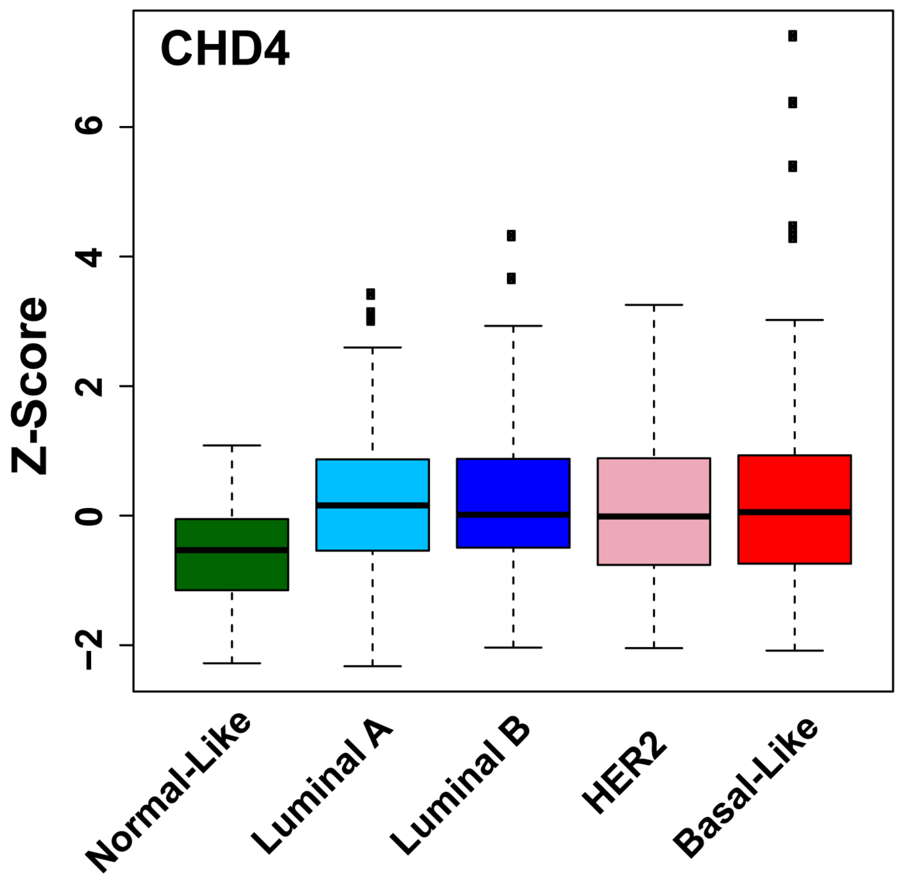

Supplementary Figure 10: Expression levels of CHD4 across five subtypes of TCGA breast cancer samples.

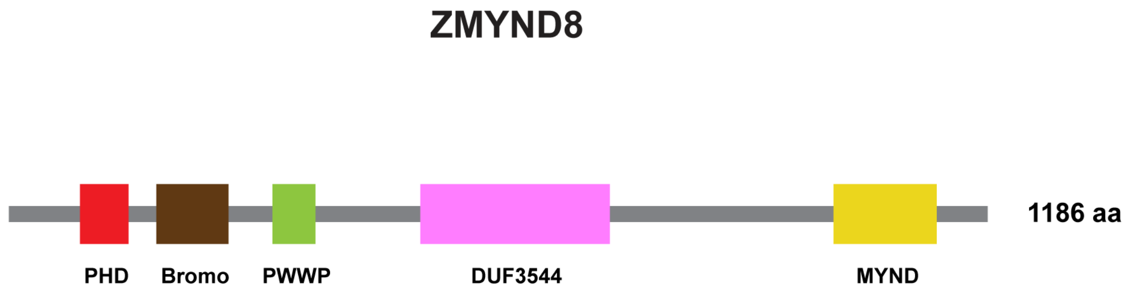

Supplementary Figure 11: Schematic structure and functional domains of ZMYND8 protein based on the data from the National Center for Biotechnology Information (NCBI).

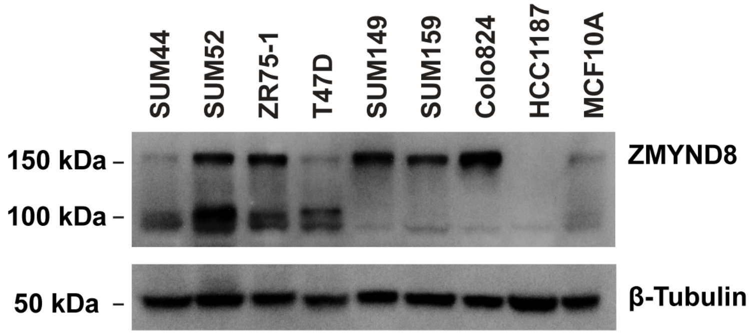

Supplementary Figure 12: Protein levels of ZMYND8 were analyzed by western blot in nine breast cancer cell lines and the MCF10A cell line. A predicted protein size of full-length ZMYND8 is approximately 131 kDa. Luminal breast cancer lines SUM44, SUM52, ZR75-1, and T47D show stronger protein bands with molecular weight lower than 100 kDa, compared with those in basal-like breast cancer lines, SUM149, SUM159, Colo824, and HCC1187.

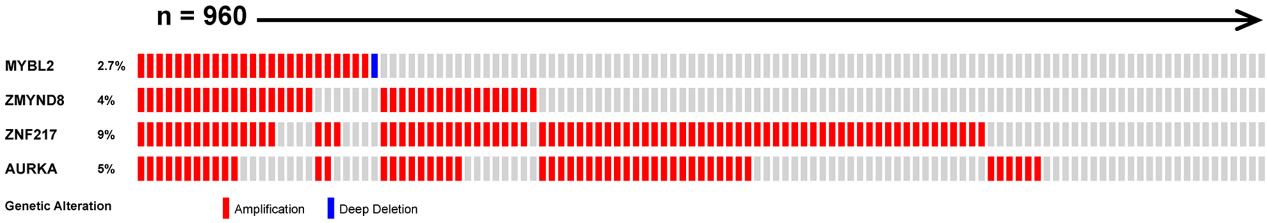

Supplementary Figure 13: High-level amplification and homozygous deletion of *ZMYND8* and other candidate oncogenes (*MYBL2*, *ZNF217,* and *AURKA*) at the 20q13 region in the TCGA breast cancer dataset (n=960). Data are displayed using the Oncoprint tool from cBioPortal.

Supplementary Table 1: List of human PHD finger-containing proteins.

See Supplementary File 1

Supplementary Table 2: Frequency (%) of genetic alterations and expression levels of PHFs in TCGA breast cancers.

See Supplementary File 2

Supplementary Table 3: Correlation between copy number and mRNA expression of PHFs in TCGA breast cancers.

See Supplementary File 3

Supplementary Table 4: CNA frequency of PHF genes in Normal-like, Luminal A, Luminal B, HER2, and Basal subtypes of TCGA breast cancers.

See Supplementary File 4

Supplementary Table 5: Expression levels of PHF genes in Normal-like, Luminal A, Luminal B, HER2, and Basal subtypes of TCGA breast cancers.

See Supplementary File 5

Supplementary Table 6: Frequency of copy number alterations of PHFs in METABRIC breast cancers.

See Supplementary File 6

Supplementary Table 7: PHF genes in 32 significantly amplified regions and 13 deleted regions of METABRIC dataset.

See Supplementary File 7

Supplementary Table 8: Mean expression levels of each PHF gene between tumor and non-tumor breast tissues in METABRIC dataset.

See Supplementary File 8

Supplementary Table 9: Mutation ratio and mutation number in PHD domain of PHF genes in TCGA breast cancers.

See Supplementary File 9

Supplementary Table 10: Mutations of ASXL1, ASXL2 and ASXL3 in TCGA breast Cancers.

See Supplementary File 10

Supplementary Table 11: Mutations of CHD3, CHD4 and CHD5 in TCGA breast Cancers.

See Supplementary File 11

Supplementary Table 12: Expression levels of PHF genes associated with AJCC tumor stages in TCGA breast cancer.

See Supplementary File 12

Supplementary Table 13: Expression levels of PHF genes associated with AJCC tumor stages in TCGA Luminal breast cancer.

See Supplementary File 13

Supplementary Table 14: Summary of log-rank statistical analysis of overall survival for PHF expression in TCGA breast cancer.

See Supplementary File 14

Supplementary Table 15: Summary of log-rank statistical analysis of overall survival for PHF expression in Luminal TCGA breast cancer.

See Supplementary File 15

Supplementary Table 16: shRNA dropout rate of 11 most amplified/gained PHF genes in 29 breast cancer cell lines based on the GARP (Gene Activity Ranking Profile) scores in COLT-Cancer database.

See Supplementary File 16
