# Supplementary material for: Integrative genomic and transcriptomic analysis for pinpointing recurrent alterations of plant homeodomain genes and their clinical significance in breast cancer
Source: Oncotarget. 2016 Dec 31;8(8):13099–115. doi: 10.18632/oncotarget.14402 (PMC5355080; doi:10.18632/oncotarget.14402)
Supplement: Supplementary file 11 [file oncotarget-08-13099-s011.xlsx]

Integrative genomic and transcriptomic analysis for pinpointing recurrent alterations of plant homeodomain genes and their clinical significance in breast cancer

Huimei Yu[1,2], Yuanyuan Jiang[1], Lanxin Liu[1], Wenqi Shan[1], Xiaofang Chu[1], Zhe Yang[3] Zeng-Quan Yang[1,4]

[1]Department of Oncology, Wayne State University School of Medicine, Detroit, MI 48201, USA

[2]College of Basic Medicine, Jilin University, Changchun, China

[3]Department of Biochemistry and Molecular Biology, Wayne State University School of Medicine, Detroit, MI 48201, USA

[4]Molecular Therapeutics Program, Barbara Ann Karmanos Cancer Institute, Detroit, MI 48201, USA

Correspondence to: *Zeng-Quan Yang,* ***email:*** *yangz@karmanos.org*

Keywords: *plant homeodomain, breast cancer, histone modification, copy number alteration*

Received: *June 16, 2016* ***Accepted:*** *December 05, 2016* ***Published:*** *December 31, 2016*

ABSTRACT

A wide range of the epigenetic effectors that regulate chromatin modification, gene expression, genomic stability, and DNA repair contain structurally conserved domains called plant homeodomain (PHD) fingers. Alternations of several PHD finger-containing proteins (PHFs) due to genomic amplification, mutations, deletions, and translocations have been linked directly to various types of cancer. However, little is known about the genomic landscape and the clinical significance of PHFs in breast cancer. Hence, we performed a large-scale genomic and transcriptomic analysis of 98 PHF genes in breast cancer using TCGA and METABRIC datasets and correlated the recurrent alterations with clinicopathological features and survival of patients. Different subtypes of breast cancer had different patterns of copy number and expression for each PHF. We identified a subset of PHF genes that was recurrently altered with high prevalence, including *PYGO2* (*pygopus family PHD finger 2*), *ZMYND8* (*zinc finger, MYND-type containing 8*), *ASXL1* (*additional sex combs like 1*) and *CHD3* (*chromodomain helicase DNA binding protein 3*). Copy number increase and overexpression of ZMYND8 were more prevalent in Luminal B subtypes and were significantly associated with shorter survival of breast cancer patients. ZMYND8 was also involved in a positive feedback circuit of the estrogen receptor (ER) pathway, and the expression of ZMYND8 was repressed by the bromodomain and extra terminal (BET) inhibitor in breast cancer. Our findings suggest a promising avenue for future research—to focus on a subset of PHFs to better understand the molecular mechanisms and to identify therapeutic targets in breast cancer.

INTRODUCTION

Histone modifications, such as methylation and acetylation, play critical roles in chromatin function, transcriptional regulation, genomic stability, and DNA repair [1, 2]. These epigenetic modifications are mediated by sets of enzymatic complexes that have complementary but opposing functions, namely the “writers,” which catalyze methylation and acetylation in a site-specific manner, and the “erasers,” which remove the modification marks [1, 2]. Such modification marks are interpreted by “reader” proteins that recognize and are recruited to the modified histone [3, 4]. One of the largest families of epigenetic effectors capable of “reading” post-translationally modified or unmodified histone tails consists of plant homeodomain (PHD) fingers. They recruit various nuclear complexes to chromatin and stabilize them [3, 5]. Among PHD finger-containing proteins (PHFs), the PHD finger exists singly or in multiple copies, in the absence of or in conjunction with other functional modules, such as distinct histone “reading” domains, e.g., bromo-, chromo-, and Tudor domains, or a catalytic

histone-associating module, such as ATPase, SET (Suppressor of variegation, Enhancer of zeste, Trithorax), and Jumonji C domains [4, 5]. PHFs can be broadly divided into the following three subgroups based on their additional functional domains and biological roles: "Epigenetic Writers," including histone methyltransferases and acetyltransferases; "Epigenetic Erasers," including histone demethylases; and "Epigenetic Readers." Thus, PHFs are vital players in regulating and maintaining the physiological functioning of epigenetic modifications in a highly context-dependent manner. Consequently, when PHFs malfunction, they are implicated in a broad range of human diseases, including cancer.

Breast cancer is the most common malignant disease in women, with more than 240,000 new cases diagnosed and 40,000 deaths in the United States per year. This heterogeneous disease is categorized into five molecular subtypes: Luminal A, Luminal B, epidermal growth factor receptor 2–enriched (HER2+), basal-like, and normal-like breast cancers [6, 7]. The majority of breast cancers (~70%) belong to the Luminal (A and B) subtypes, characterized by expression of the estrogen receptor-α (ERα). ERα is the principal biomarker for directed hormone therapies and is the primary therapeutic target in breast cancer [8]. Luminal B breast cancers have lower expression of ERα and higher histologic grade, and they are less responsive to hormone therapy and have poorer outcomes than Luminal A [9]. Furthermore, basal-like breast cancer usually occurs in young women and is a highly aggressive subtype associated with very poor prognosis [10]. By deeply understanding the genetic and epigenetic alterations that are associated with the different types of breast cancer, we can identify new druggable subtype-specific targets for effective therapies.

Recent studies revealed that tumors, including breast cancer, have frequent genetic alterations in histone modifiers, including PHFs [18-20]. PHD fingers have been shown to play a critical role in oncogenic drivers; for example, a chromosomal translocation in the PHD finger of PHF23 or lysine (K)-specific demethylase 5A (KDM5A) was implicated in acute myeloid leukemia [11, 12]. We demonstrated that KDM4C and KDM5A are frequently amplified and overexpressed in breast cancer, particularly in aggressive basal-like subtypes [13, 14]. The PHD fingers in histone lysine methyltransferase WHSC1 (Wolf-Hirschhorn syndrome candidate 1) are critical for recruiting WHSC1 to oncogenic gene loci and driving multiple myeloma [15]. WHSC1L1, a homolog of WHSC1, is significantly amplified and overexpressed in a subset of breast, lung, and pancreatic cancers [16, 17]. Furthermore, the bromodomain PHD finger transcription factor (BPTF) is amplified and overexpressed in melanomas, and BPTF is required for c-MYC transcriptional activity and *in vivo* tumorigenesis [18]. In contrast, a *chromodomain helicase DNA binding 5* (*CHD5*) gene, which encodes an ATPase-dependent DNA-binding protein with two PHDs, is a tumor suppressor in neuroblastomas. PHD-mediated histone 3 binding is required for CHD5-mediated tumor suppression [19, 20]. In addition, the *additional sex combs like 1* (*ASXL1*) is one of the most frequently mutated genes in malignant myeloid diseases, and *ASXL1* mutations are strongly associated with a poor prognosis in these myeloid disorders [21, 22].

The initiation and progression of hematological malignancies and solid tumors have been associated with dysregulation of several PHFs. However, little is known about the rest of the genomic landscape and the clinical significance of PHFs in breast cancer. Thus, we performed a comprehensive, integrated genomic and transcriptomic analysis of 98 PHF genes in breast cancer and identified associations among recurrent copy number alteration, gene expression, clinicopathological features, and survival of patients. This approach enabled us to identify a subset of PHF genes that were recurrently altered with high prevalence, such as *PYGO2* (*pygopus family PHD finger 2*), *ZMYND8* (*zinc finger and MYND [myeloid, Nervy, and DEAF-1] domain containing 8*), *ASXL1,* and *CHD3* (*chromodomain helicase DNA binding protein 3*). High expression of ZMYND8 was significantly correlated with patient survival and was likely involved in a positive feedback circuit of the ER pathway in breast cancer. Furthermore, we found that JQ-1, a bromodomain and extra terminal (BET) inhibitor, suppressed ZMYND8 expression in breast cancer. These findings prioritize a subset of PHFs for future research focused on understanding molecular mechanisms and therapeutic potentials in breast cancer.

RESULTS

Copy number and expression profiling of PHFs in breast cancer

Genetic alterations, including copy number alteration (CNA) and somatic mutation, are a universal hallmark of cancer [23, 24]. We hypothesized that PHFs with recurrent genetic alterations might play important roles in different types of breast cancer and hence serve as novel therapeutic targets. Based on the current ChromoHub database, there are 99 PHFs in the human genome (Supplementary Figure 1 and Table 1) [25]. We first analyzed CNAs and mutations in 98 PHF genes (excluding *KDM5D* on chromosome Yq11) compiled from 960 breast cancer specimens in The Cancer Genome Atlas (TCGA) *via* cBioPortal [26, 27]. The copy number for each PHF was generated by the copy number analysis algorithm GISTIC (Genomic Identification of Significant Targets in Cancer) and categorized, as copy number level per gene, into the following: high-level amplification, low-level gain, diploid, heterozygous deletion, and homozygous deletion [26, 27]. We first grouped the copy number of each PHF gene of TCGA breast cancer samples into amp/gain

(high-level amplification and low-level gain), diploid, and deletion (heterozygous or homozygous deletions). As shown in Table 1 and Supplementary Table 2, the 11 most frequently (>40%) amplified/gained PHF genes were *KDM5B*, *ASH1L*, *PYGO2*, *PHF20L1*, *CREBBP*, *FBXL19*, *DIDO1*, *ZMYND8*, *PHF20*, *ASXL1*, and *BPTF*; and the 9 most frequently (>40%) deleted genes were *PHF23*, *CHD3*, *RAI1*, *KMT2A*, *TCF20*, *PHF21B*, *PHF11*, *EP300*, and *BRD1*. The most frequently (>1.5%) mutated PHF genes were *KMT2C*, *KMT2D*, *CHD4*, *ASH1L*, *ASXL3*, *ASXL2*, *KMT2A,* and *BAZ2B*. Notably, *ASH1L* exhibited a higher frequency of both amp/gain (73.93%) and mutation (1.77%), whereas *CHD3* and *KMT2A* showed higher frequency of both genetic deletion (61% and 49.01%, respectively) and mutation (1.46% and 1.67%, respectively) in breast cancer (Table 1 and Supplementary Figure 2).

We next examined the mRNA expression levels of each PHF in TCGA breast cancer samples. As shown in Table 1 and Supplementary Table 2, we found that three PHFs (*PYGO2*, *KDM5B*, and *PHF20L1*) were overexpressed at the mRNA level (Z-score > 1) in more than 40% of breast cancers. *PYGO2* had the highest frequency (51.41%) of mRNA overexpression. In contrast, the most deleted gene, *PHF23,* had the highest frequency (43.48%) of mRNA underexpression (Z-score < -1), and *CHD3* was underexpressed in 35.14% of TCGA breast cancer samples. We also analyzed the correlation between copy number and mRNA level of 97 PHFs (excluding those of *KDM5D* and *KMT2B,* as their RNA-sequencing data were not available) from TCGA breast cancer specimens. As shown in Supplementary Table 3, almost all PHF genes had positive correlations between DNA copy number and mRNA expression, and 24 of them had a Spearman correlation coefficient (r) > 0.5.

To determine whether the genetic alteration or mRNA expression of each PHF is specific to a breast cancer subtype, we analyzed CNA and mRNA expression independently across different subtypes of 808 breast cancer samples for which PAM50 (Prediction Analysis for Microarray 50) subtype data were available [26]. The frequencies of high-level amplification, low-level gain, diploid, heterozygous deletions, homozygous deletions, and somatic mutation of PHF genes in five breast cancer subtypes are shown in Supplementary Table 4. mRNA expression status of the PHF genes is shown in Supplementary Table 5. Among the 11 most amplified/gained PHF genes, 7 (*PYGO2, KDM5B*, *PHF20L1*, *ASH1L*, *PHF20*, *DIDO1*, and *ASXL1*) likely had a higher frequency of gain/amplification in both Luminal and basal subtypes of breast cancer, and 4 of them (*ZMYND8*, *BPTF*, *CREBBP*, and *FBXL19*) were more commonly amplified/gained in Luminal, particularly Luminal B breast cancer, than in the normal-like subtype (Figure 1A, Supplementary Table 4). Among the 9 most deleted PHF genes, *PHF23*, *CHD3*, *RAI1,* and *PHF11* had more deletions in Luminal, HER2+, or basal-like subtypes, while *KMT2A*, *TCF20*, *PHF21B*, *EP300,* and *BRD1* had more deletions in the Luminal B subtype (Figure 1B, Supplementary Table 4). A detailed analysis of expression levels of each PHF in the five breast cancer subtypes also revealed that expression levels of *PYGO2* and *KDM5B* were higher in Luminal, HER2+, and basal-like, compared with their expression levels in the normal-like subtype of breast cancer (Figure 1, Supplementary Figure 3, and Supplementary Table 5). We also found that *ZMYND8* has a higher expression level in Luminal and HER2+, but not in the basal-like subtype, compared with that in the normal-like subtype breast cancer (Figure 1). In contrast, the commonly deleted genes *CHD3* and *KMT2A* showed underexpression in HER2+ and basal-like subtypes (Figure 1, Supplementary Table 5).

To validate our findings from TCGA breast cancer dataset regarding PHF genetic alterations, we conducted an independent analysis using the METABRIC dataset, which contains approximately 2000 primary breast cancers with long-term clinical follow-up data. We found that 17 PHF genes, including *PYGO2*, *KDM5B*, *PHF20L1*, and *ZMYND8*, had a higher frequency (>10%) of gain/amplification, and 9 PHF genes, including *CHD3* and *PHF23*, had a higher frequency of deletion in the METABRIC breast cancer samples (Supplementary Table 6), although the frequency of gain/amplification identified in the METABRIC dataset is lower than that of TCGA dataset, possibly due to the different CNA analysis platforms and calling algorithms. Furthermore, statistical analyses of copy number alterations in METABRIC dataset define 32 regions of amplification and 13 regions of deletion [28]. We analyzed these 45 genomic regions and found that 11 PHF genes are localized in significantly amplified regions, including *KDM5A* in 1q32, *WHSC1L1* and *ASH2L* in 8p11-12, *PHF20L1* in 8q13-24, and *ZMYND8* in 20q13 (Supplementary Table 7). We also found that Luminal B breast cancer had the highest frequency of *ZMYND8* gain/amplification in the METABRIC dataset (Supplementary Figure 4A). Expression levels of PYGO2, KDM5B, PHF20L1, and ZMYND8 were also significantly higher in tumor samples compared to that in non-tumor breast tissue (Supplementary Table 8). Again, mRNA expression levels of PYGO2 and KDM5B were higher in Luminal, HER2+, and basal-like breast cancers, and *ZMYND8* was higher in Luminal B and HER2+ subtypes compared with that in the normal-like subtype in the METABRIC dataset (Supplementary Figure 4B; $p < 0.001$).

Recurrent mutations of CHD3-5 and ASXL1-3 genes in breast cancer

Among mutated PHF genes in TCGA breast cancer samples, we found that 33 PHF genes contained mutations in the PHD domains (Supplementary Table 9). We noticed

Table 1: Frequency (%) of genetic and transcriptional alterations of PHFs that are highly prevalent in TCGA breast cancers

Gene	Location	DNA Alterations				mRNA Expression Levels		
		Amp/Gain	Diploid	Deletion	Mutation	Z Score >= 1	1>Z Score > -1	Z Score <= -1
PYGO2	1q21.3	73.62	25.03	1.36	0.10	51.41	41.92	6.67
KDM5B	1q32.1	75.81	22.63	1.56	1.25	46.09	48.80	5.11
PHF20L1	8q24.22	59.65	35.56	4.80	0.73	41.40	52.03	6.57
ASH1L	1q22	73.93	24.50	1.56	1.77	37.96	51.51	10.53
CREBBP	16p13.3	53.49	38.16	8.34	1.46	32.64	55.16	12.20
BPTF	17q24.2	41.50	46.40	12.10	1.36	32.01	53.81	14.18
DIDO1	20q13.33	48.38	47.13	4.48	0.94	31.60	55.16	13.24
PHF20	20q11.22-q11.23	44.11	50.89	5.01	0.31	27.53	59.12	13.35
ZMYND8	20q13.12	46.72	48.80	4.48	0.52	19.60	69.45	10.95
FBXL19	16p11.2	52.03	39.62	8.34	0.21	19.29	70.91	9.80
ASXL1	20q11.21	42.44	52.87	4.69	0.52	27.53	58.29	14.18
ASXL2	2p23.3	14.91	67.15	17.94	1.67	17.62	64.03	18.35
ASXL3	18q12.1	15.33	55.89	28.78	1.67	2.19	97.81	0.00
CHD3	17p13.1	5.53	33.47	61.00	1.46	6.26	58.60	35.14
CHD4	12p13.31	25.23	60.38	14.39	2.09	19.29	64.65	16.06
CHD5	1p36.31	7.30	53.28	39.42	1.46	1.67	98.33	0.00
KMT2A	11q23.3	9.38	41.61	49.01	1.67	10.74	63.82	25.44
KMT2C	7q36.1	24.71	55.47	19.81	6.99	15.95	68.72	15.33
KMT2D	12q13.12	20.13	65.69	14.18	2.40	17.62	64.23	18.14
BAZ2B	2q24.2	8.45	69.86	21.69	1.56	12.41	69.03	18.56
BRD1	22q13.33	12.20	42.54	45.26	0.63	11.05	55.89	33.06
EP300	22q13.2	10.32	44.00	45.67	1.15	12.20	64.13	23.67
PHF11	13q14.2	7.61	46.30	46.09	0.31	9.38	61.21	29.41
PHF21B	22q13.31	11.37	42.34	46.30	0.31	3.86	96.14	0.00
TCF20	22q13.3; 22q13.2	10.84	42.75	46.40	1.46	12.10	62.46	25.44
RAI1	17p11.2	7.92	37.02	55.06	0.42	9.28	59.54	31.18
PHF23	17p13.1	5.11	33.37	61.52	0.21	6.67	49.84	43.48

that three PHF subfamilies (*KMT2A/C/D*, *ASXL1-3*, and *CHD3-5*) had higher frequencies of mutation. We also analyzed frequencies of mutation number per gene size, and found that the higher frequent mutation ratios were associated with *KMT2C*, *CHD4*, *ASXL2,* and *BAZ2B*, which have mutations of more than 2 per kilobase (kb) (Supplementary Table 9). We previously reported a mutation spectrum for *KMT2C* and *KMT2D* and proposed their function as tumor suppressors in breast cancer [29]. Here, we focused on analyzing the mutation spectrum of *ASXL1-3* and *CHD3-5* genes in breast cancer.

The ASXL family consists of three members (ASXL1, ASXL2 and ASXL3) that share a common domain architecture: HARE-HTH, ASXH, and a C-terminal PHD. As shown in Figure 2A and Supplementary Table 10, we identified a total of 40 ASXL family mutations, consisting of 7 mutations of *ASXL1*, 17 mutations of *ASXL2*, and 16 mutations of *ASXL3*. One tumor sample (TCGA-BH-A0B9)

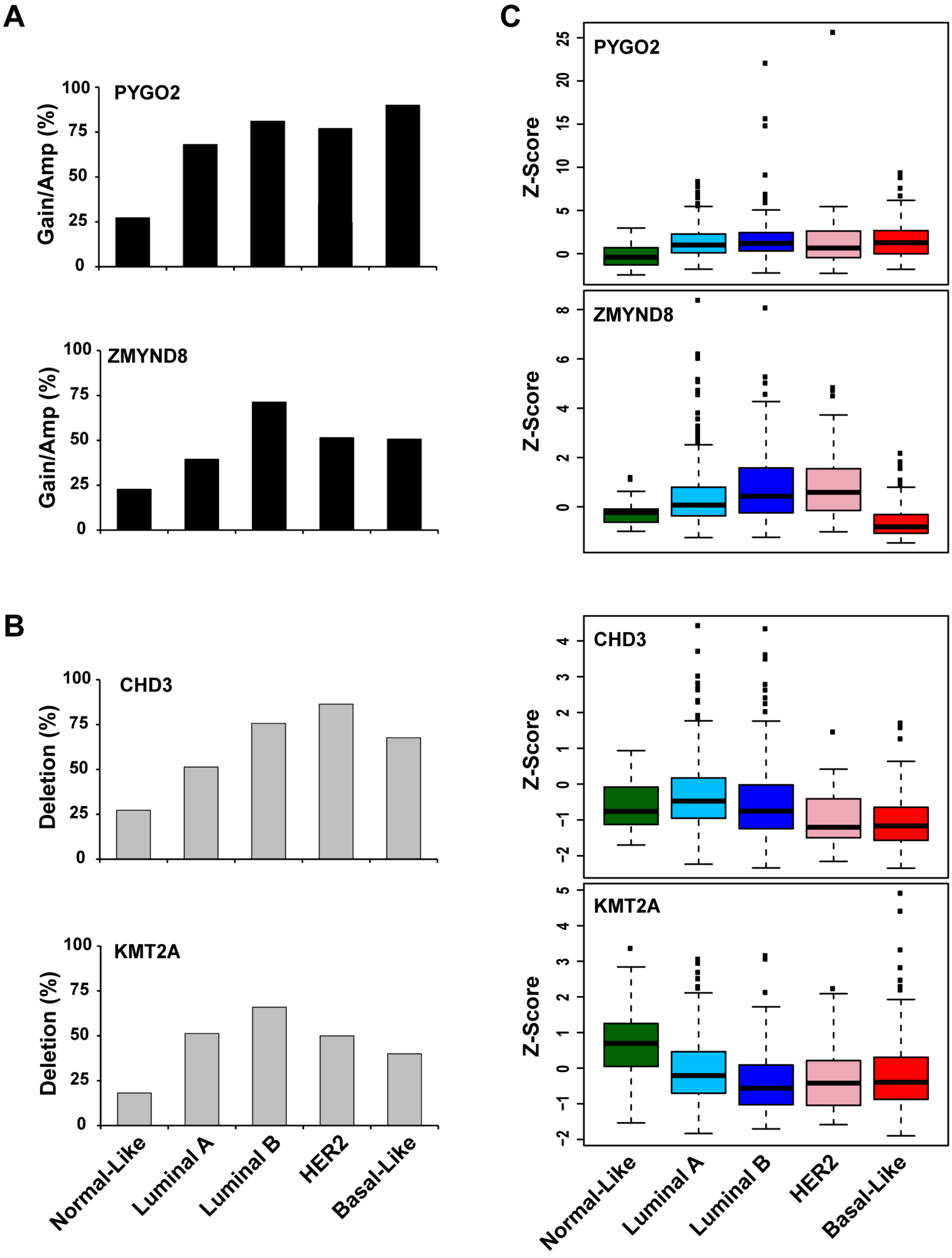

Figure 1: Frequencies of *PYGO2* and *ZMYND8* copy number increase **A.**, and *CHD3* and *KMT2A* deletion **B.** across five subtypes of TCGA breast cancer samples. **C.** Expression levels of *PYGO2*, *ZMYND8*, *CHD3*, and *KMT2A* across five subtypes of TCGA breast cancer samples. The differences in *PYGO2*, *ZMYND8*, *CHD3*, and *KMT2A* mRNA levels among breast cancer subtypes are statistically significant ($p < 0.05$).

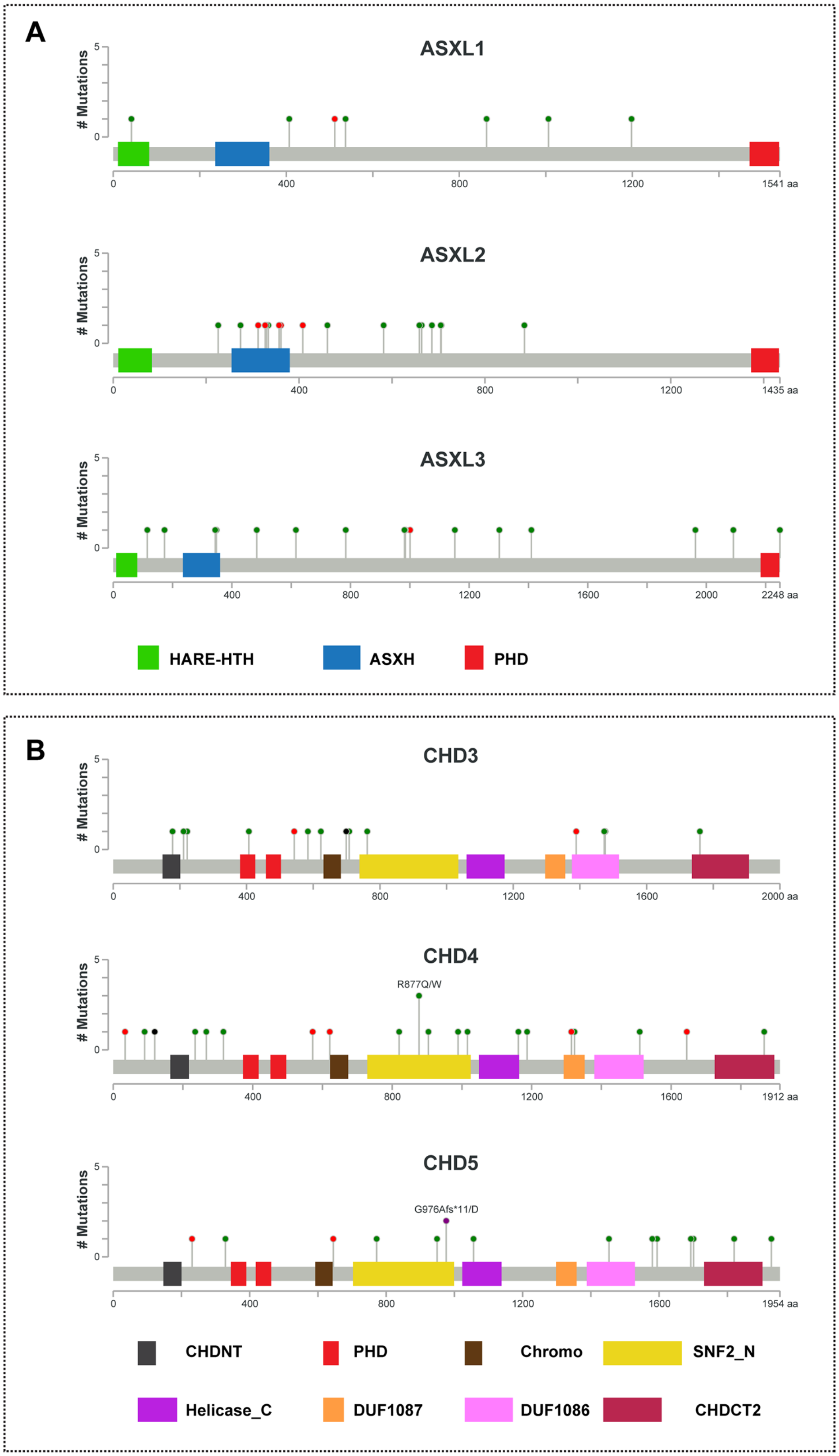

Figure 2: Mutational spectra of ASXL1-3 and CHD3-5 genes in breast cancer. The images show protein domains and the positions of somatic mutations in ASXL1-3 **A.** and CHD3-5 **B.** in TCGA breast cancers. A red dot indicates a nonsense mutation, frameshift deletion, insertion, or splice; a green dot indicates a missense mutation; and a black dot indicates an inframe insertion or deletion. The data were obtained from TCGA database via cBioPortal.

had three missense mutations (D893H, E1006K, and G1198V) in the *ASXL1* gene, and one sample (TCGA-AN-A046) had two nonsense mutations (R357* and R312*) in the *ASXL2* gene. Most of the mutations in the *ASXL* genes are localized to the amino-terminal end of the PHD domain. Previous studies demonstrated that truncation mutations of *ASXLs* occur in autism, Bohring–Opitz and related syndromes, hematological malignancies, and several solid tumors [21, 22, 30]. Therefore, we predict that mutations at the amino terminus of the PHD might result in the gain or loss of function of ASXL proteins in breast cancer.

CHD3, CHD4, and CHD5 belong to the second subfamily of CHD proteins, which are characterized by an SNF2-like domain located in the central region as well as tandem PHD and chromodomains at their C-termini [31, 32]. The SNF2-like domain is responsible for ATP-dependent chromatin remodeling. As shown in Figure 2B and Supplementary Table 11, we identified a total of 51 CHD3-5 family mutations, consisting of 14 mutations of *CHD3*, 22 mutations of *CHD4,* and 15 mutations of *CHD5*, most of which were missense mutations. In the *CHD4* gene, tumor sample TCGA-C8-A27B had two missense mutations (P90Q and A235P), and TCGA-D8-A1JN had one missense mutation (I989F) and an X34 splice. In the *CHD5* gene, one sample (TCGA-D8-A1JK) had a missense mutation (H1820Y) and frameshift deletion (G976Afs*11). Furthermore, we found that one sample (TCGA-BH-A1FC) had a mutation (D407H) in the region of the first PHD-finger of CHD3 (Figure 2B).

Association of PHF gene expression with clinical features and survival of breast cancer patients

To investigate the clinical relevance of PHF alterations in breast cancer, we examined expression levels of each PHF gene at different stages of TCGA breast cancer samples. The means of Z-score and *p*-value for each PHF gene across four American Joint Committee on Cancer (AJCC) stages of breast cancer are shown in Supplementary Table 12. Among 11 most commonly amplified/overexpressed PHF genes, we found that three genes, *ZMYND8, PHF20*, and *DIDO1*, were significantly highly expressed in advanced-stage breast cancers (T-test: Stage I+II vs III+IV; $p < 0.05$; Figure 3A and Supplementary Table 12). Because several PHFs were more commonly amplified/gained in the Luminal subtype of breast cancer, we then examined the expression levels of each PHF gene in different stages of only Luminal breast cancer samples. We found that *ZMYND8* and *PHF20,* but not *DIDO1,* also had significantly higher expression in advanced stages of Luminal breast cancers ($p < 0.05$; Supplementary Table 13).

Next, to analyze the relationship between PHF mRNA expression and overall survival of breast cancer patients, samples were divided into high (Z-score > 0) and low (Z-score =< 0) groups based on the mRNA expression level of each PHF. Supplementary Table 14 summarizes the results of a log-rank statistical analysis of 97 PHFs in breast cancer. High mRNA levels of *ZMYND8*, *BPTF*, *PHF20*, *WHSC1L1*, and *PHF20L1* were positively associated with shorter survival in breast cancer patients ($p < 0.05$) (Figure 3B, Supplementary Table 14). We also performed survival analyses of each PHF gene in Luminal (A and B) subtypes only. In the Luminal subtype, we found that high mRNA levels of six PHF genes, *PHF20*, *PYGO1*, *KDM5A*, *PHF6*, *BPTF*, and *CHD5*, were significantly associated ($p < 0.05$) with shorter survival in breast cancer patients. Conversely, low mRNA levels of *KDM4B* and *ING* were significantly associated ($p < 0.05$) with shorter survival (Supplementary Table 15). We then performed a multivariate analysis to investigate whether the expression level of each PHF was predictive of poor prognosis compared with standard prognostic markers, including age at diagnosis, ER status, progesterone receptor status, HER2 status, tumor size, lymph node status, metastasis status, and molecular subtype (basal vs. non-basal). In addition to *PHF20L1*, which we previously reported, we also found that high mRNA level of *ZMYND8* ($p = 0.034$, hazard ratio [HR] = 1.92) was independently associated with shorter survival of TCGA breast cancer patients (Figure 3C) [33]. Validating this analysis using the METABRIC dataset, we found that *ZMYND8* was similarly highly expressed in breast cancer samples of advanced stage and higher grade (Supplementary Figure 5). We confirmed that higher expression of *ZMYND8* was correlated with a poor prognosis ($p = 0.034$, HR = 1.16) in METABRIC breast cancer samples.

ZMYND8 in the feedback circuit of the ER pathway was suppressed by the BET inhibitor

In our analysis of the genetic alterations of PHFs in breast cancer, we found that ZMYND8 had a higher frequency of amplification and overexpression in Luminal B breast cancer, and its overexpression was associated with shorter survival in patients in both TCGA and METABRIC datasets. We further examined ZMYND8 expression in a panel of breast cancer cells. Quantitative reverse transcription PCR (qRT-PCR) and RNA sequencing showed that ZMYND8 was expressed more in ER-positive Luminal than ER-negative basal cell lines (Figure 4A and Supplementary Figure 6). Because a previous study demonstrated that ZMYND8 physically binds ERα in breast cancer [34], and because we found that mRNA levels of ZMYND8 were positively correlated with expression levels of the *ESR1* (estrogen receptor 1) gene (Spearman correlation coefficient (r) of 0.34 in TCGA breast cancer samples), we tested whether ZMYND8 is a downstream target of the ER pathway. We treated two ER-positive Luminal breast cancer cell lines, T47D and ZR75-1, with tamoxifen, the most widely used nonsteroidal selective ER modulator for adjuvant therapy of ER-

A

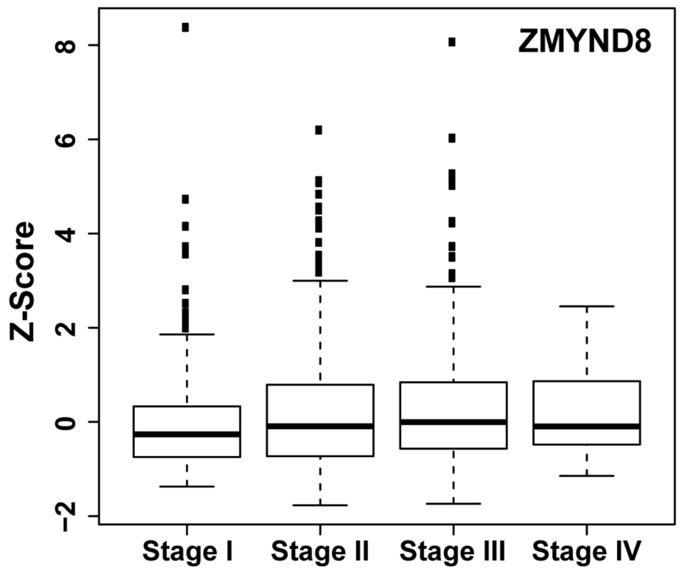

B

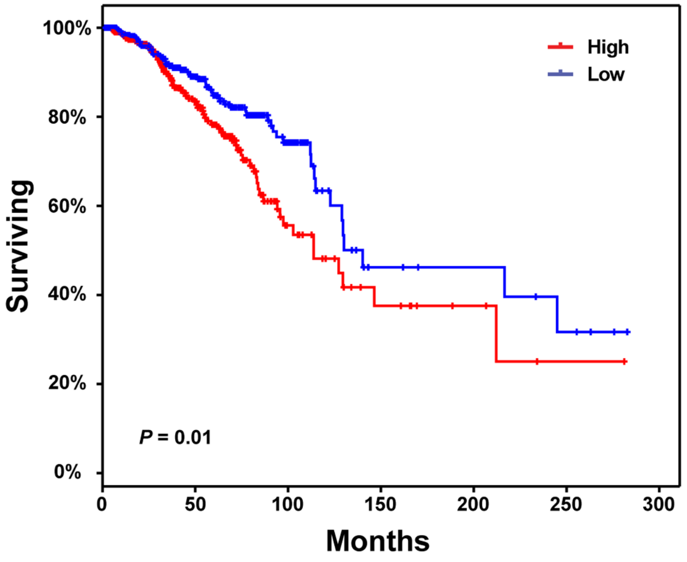

C

	p value	HR	95% CI
ZMYND8 expression	0.034	1.916	1.049-3.497
Age , continuous	0.004	1.029	1.009-1.050
ER, positive versus negative	0.300	0.609	0.238-1.556
PR, positive versus negative	0.003	0.321	0.151-0.683
HER2, positive versus negative	0.043	0.363	0.136-0.968
pT, PT2-3 versus pT1	0.516	1.259	0.629-2.519
pN, positive versus negative	0.192	1.465	0.825-2.602
Metastasis, positive versus negative	0.067	2.290	0.943-5.558
PAM50, basal versus nonbasal	0.247	0.570	0.220-1.476

Figure 3: A. Expression levels of *ZMYND8* across different AJCC stages of TCGA breast cancer samples. **B.** Kaplan-Meier plots of overall survival associated with mRNA expression levels of *ZMYND8* in TCGA breast cancers. **C.** Multivariate analysis revealed that *ZMYND8* expression was independent of prognostic variables influencing overall survival of TCGA breast cancer patients.

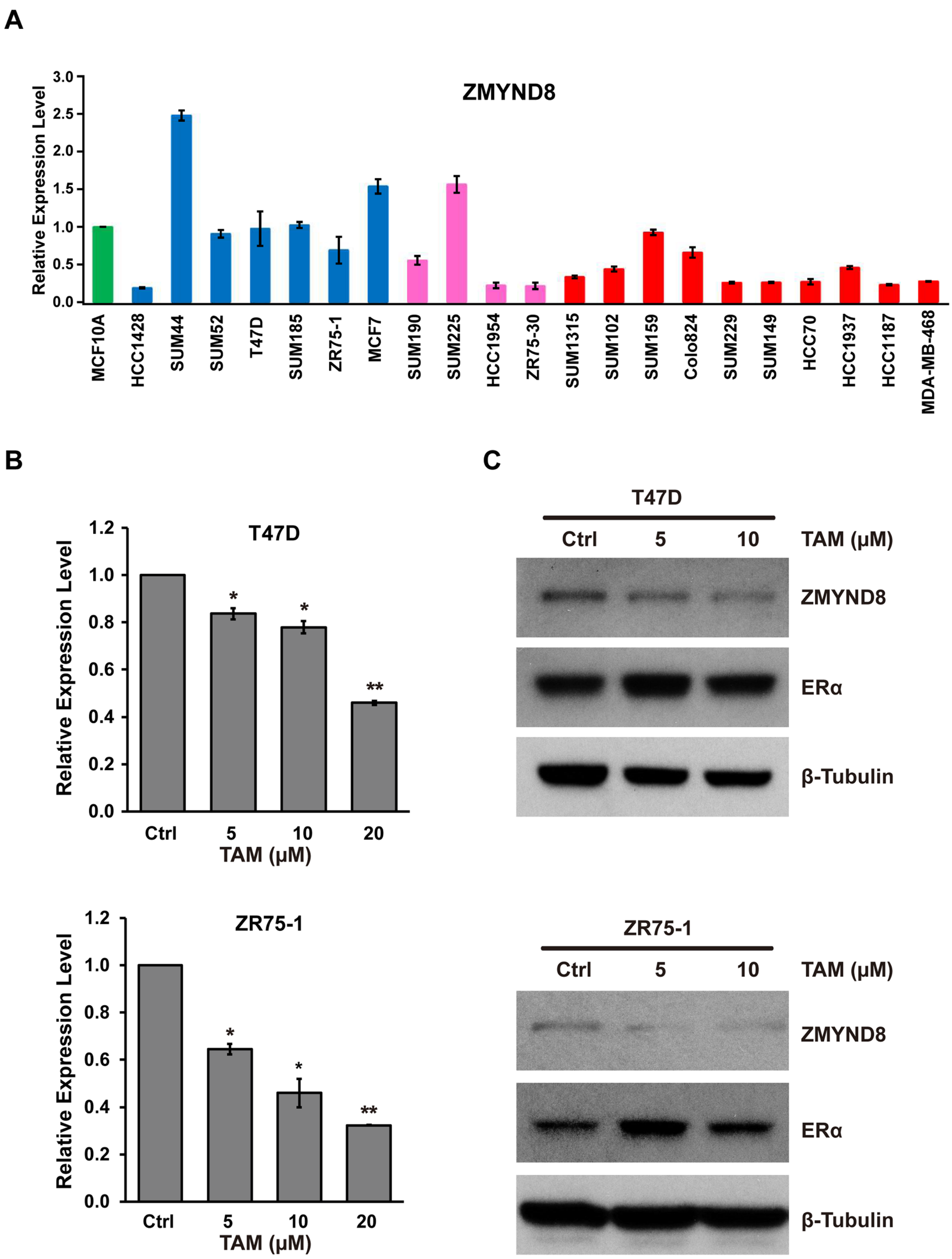

Figure 4: A. mRNA expression levels of *ZMYND8*, measured by qRT-PCR, in a panel of 21 breast cancer cell lines. mRNA expression levels in the immortalized but nontumorigenic breast epithelial cell line MCF10A cells were arbitrarily set as 1. Cell lines: green indicates MCF10A; blue, Luminal breast cancer cell lines; pink, HER2+ breast cancer cell lines; and red, basal-like breast cancer cell lines. **B.** qRT-PCR and **C.** immunoblot analysis of ZMYND8 expression after treatment with tamoxifen (TAM) in T47D and ZR75-1 cells (*$p < 0.05$ and **$p < 0.01$, Student's *t*-test). Protein levels of ERα were also measured by western blot after TAM treatment.

positive breast cancer. Supplementary Figure 7 shows that expressions of ERα target genes *TFF1* (*trefoil factor 1*) and *MYB* were suppressed in T47D cells after tamoxifen treatment. We found that expression of ZMYND8 at mRNA and protein levels was also significantly reduced in those two cell lines after tamoxifen treatment (Figure 4B and 4C). Next, we analyzed published data of ERα chromatin immunoprecipitation sequencing (ChIP-Seq) in ER-positive breast cancer cell lines [35, 36]. We found that ERα directly bound to the genomic regions of the *ZMYND8* gene. Supplementary Figure 8 illustrates ChIP-Seq binding sites of ERα at the *ZMYND8* genomic loci in T47D cells [36, 37]. Thus, these data suggest that a positive regulatory loop between ERα and ZMYND8 exists in ER-positive luminal breast cancer.

Recent studies demonstrated that bromodomain-containing protein-4 (BRD4) plays an important role in promoting estrogen-regulated transcription of ER-positive breast cancer cells [38]. BRD4 is a member of the BET family, which also includes BRD2, BRD3, and BRDT. BRD4 is a major target of BET inhibitors, such as JQ-1, which also suppress breast cancer growth inhibition *in vitro* and *in vivo* [39]. We validated that JQ-1 inhibited growth and survival of both ER-positive luminal T47D and basal-like SUM159 breast cancer cells (Figure 5A and 5B). The Western blot assays demonstrated that JQ-1 downregulated c-MYC, a known target of BRD4, but there was no measurable effect on ERα expression in T47D cells (Figure 5C and Supplementary Figure 9). We also found that JQ-1 dramatically suppressed the mRNA and protein expression of ZMYND8 in both ER-positive T47D and ER-negative SUM159 cells (Figure 5C and 5D). Thus, ZMYND8 expression is likely regulated by multiple mechanisms, including gene amplification, ERα, and possibly BRD4 pathways in breast cancer.

DISCUSSION

By conducting integrated genomic and transcriptomic analyses of breast cancers with different molecular subtypes and clinicopathological features, we identified a subset of PHF genes, including *PGYO2*, *CHD3*, and *ZMYND8*, that have high frequencies of CNA and altered mRNA expression. Different subtypes of breast cancer had different patterns of copy number and expression of each PHF. Several PHF genes, e.g. *PYGO2*, were amplified/overexpressed likely independent of subtype. Copy number increase and overexpression of ZMYND8 were more prevalent in Luminal B subtypes and were significantly associated with shorter survival of breast cancer patients. Interestingly, we found that ZMYND8, which physically binds ERα, is a downstream target of ERα, suggesting that ZMYND8 is in a positive feedback circuit of the ER pathway in breast cancer. Furthermore, the expression of ZMYND8 was repressed after treatment with the BET inhibitor JQ-1 in breast cancer cells.

The PHD finger, which is approximately 50-80 amino acids long, is a sequence-specific histone recognition protein domain [5]. The PHD finger consists of two anti-parallel β-sheets and a C-terminal α-helix, which is stabilized by two zinc atoms. Biochemically, PHD fingers are classified into canonical PHD fingers that bind to trimethylated histone H3 lysine 4 (H3K4me3) or noncanonical PHD fingers that bind to differently modified histones or DNA. H3K4me3 is a histone mark associated with active transcription. PHD binding of H3K4me3 occurs through an aromatic cage, similar to those observed in chromodomain, PWWP (Pro-Trp-Trp-Pro), and Tudor domains [4, 5]. Of note, one tryptophan (Trp) residue in the second β-strand of canonical PHD is absolutely conserved; this Trp forms one of the walls of the aromatic cage for H3K4me3 binding [5]. In contrast, noncanonical PHD fingers that bind unmodified histone H3 lack the second β-strand Trp. Among 99 PHF proteins with PHD fingers based on the current ChromoHub database, 57 proteins, including PYGO2, contain only one PHD, and most of them bind H3K4me3 (Supplementary Table 1). Thirty-two PHF proteins, e.g. CHD3-5, have two PHDs, and many of them, particularly those with two PHDs in tandem, bind unmodified histone H3, due to the lack of conserved Trp residues in both PHDs. In addition, 11 PHF proteins have more than two PHDs; one of them is histone methyltransferase KMT2C, which has eight PHDs, and likely has diverse histone-binding and biological roles [40]. Furthermore, many PHFs also have additional epigenetic effector domains, including bromo-, chromo-, Tudor, and PWWP domains. We found the coexistence of a PHD and bromodomains in 22 PHF proteins, including BPTF and ZMYND8; thus, that pairing is considerably more frequent than the pairing of any other epigenetic effector domains. Bromodomain is a “reader” of lysine acetylation [39]. A combined readout of epigenetic marks by the PHD and bromodomain has been characterized in several PHFs [4, 41]. For example, the PHD finger domain of BPTF recognizes methylation signatures at H3K4me2/3, and the bromodomain selectively binds to the H4K16ac acetylation mark [41]. Thus, structure-function studies have highlighted the fact that PHD fingers recognize histone tails with relatively high specificity and affinity, making them critical components of various epigenetic mechanisms.

In this study, we identified four PHFs, *PYGO2*, *KDM5B*, *PHF20L1,* and *ASH1L*, which were most commonly amplified/overexpressed in breast cancer (Table 1). These data agree with and consolidate prior reports on the genetic alterations and oncogenic potentials of these PHFs in various tumors. PYGO2 protein contains one PHD domain, which regulates β-catenin-mediated transcription through an interaction with methylated H3K4 marks [42, 43]. Recent studies demonstrated that PHD-mediated PYGO2 chromatin binding supports and enhances mammary gland progenitor proliferation

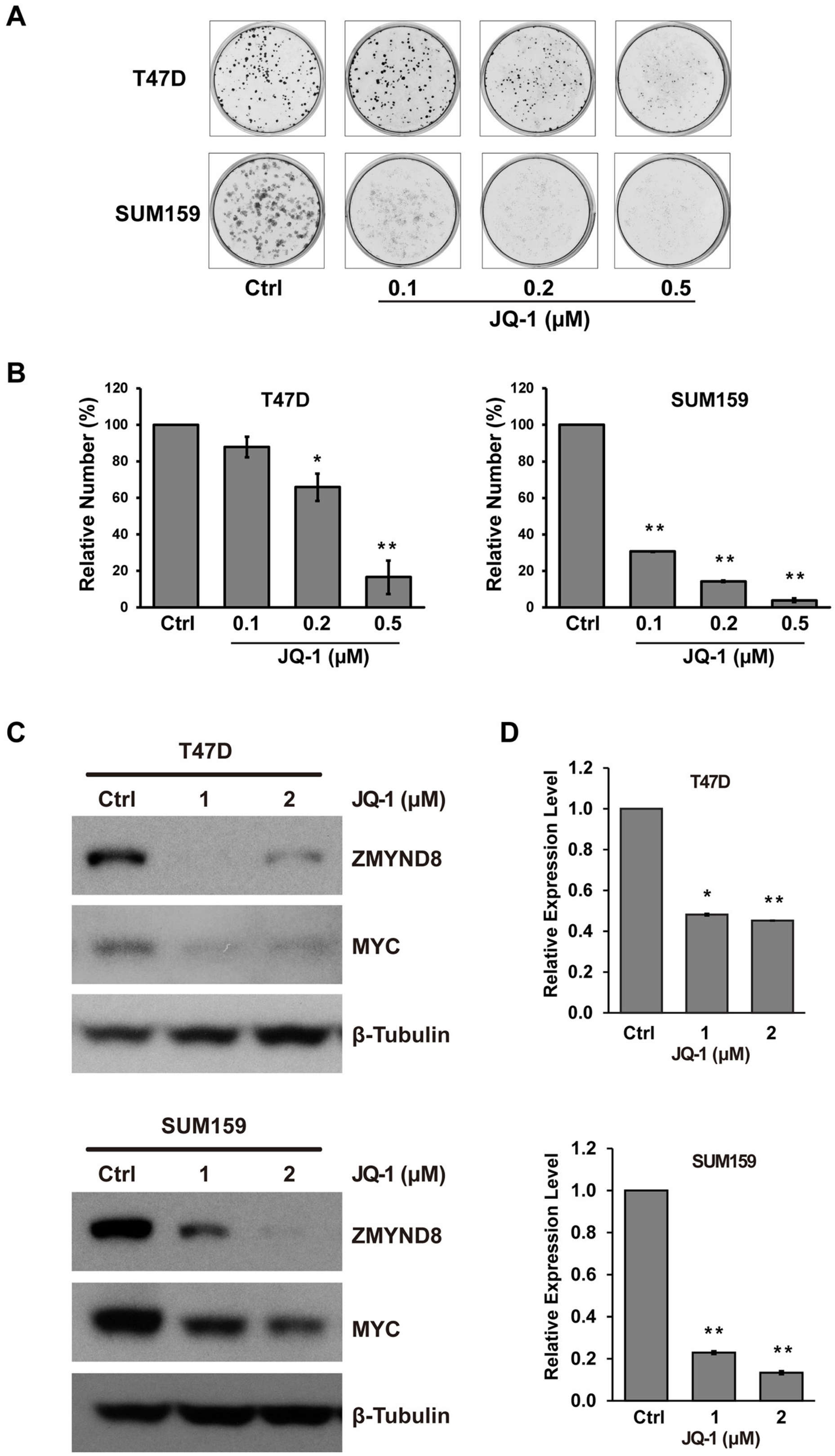

Figure 5: A. Representative images of cells stained with crystal violet show the effect of JQ-1 on growth and survival of ER-positive T47D and ER-negative SUM159 cells. **B.** Bar graphs show relative cells growth after JQ-1 treatment in T47D and SUM159 cells. **C.** Immunoblot and **D.** qRT-PCR analysis of ZMYND8 expression after treatment with JQ-1 in T47D and SUM159 cells (*p<0.05 and **p<0.01, Student's t-test). Protein levels of MYC were also measured by western blot after JQ-1 treatment in T47D and SUM159 cells.

[44, 45]. Furthermore, loss of epithelial Pygo2 delayed mammary tumor onset in mouse mammary tumor virus (MMTV)-Wnt1 transgenic mice [46]. *KDM5B* was originally discovered as a gene that was upregulated by HER2 in breast cancer cells [47]. Consistent with this, we found that the mRNA expression level of *KDM5B* was slightly higher in the HER2+ subtype, compared with luminal and basal-like subtypes, in both TCGA and METABRIC breast cancer samples (Supplementary Figure 3). Inhibiting KDM5B in breast cancer cells has been shown to reduce proliferation-reduced mammary tumor growth *in vitro* and *in vivo* [48, 49]. In addition, COLT analysis of the genes essential for cancer cell survival and proliferation in 29 breast cancer lines identified KDM5B as a hit in 12 (41%) of these lines (Supplementary Table 16) [50]. PHF20L1 protein contains two types of histone-reading modules, PHD and Tudor domains. PHF20L1 is amplified and overexpressed in a subset of basal-like and Luminal B breast cancers. We recently demonstrated that knockdown of PHF20L1 inhibits cell proliferation in breast cancer cell lines [33]. We speculate that PHF20L1 likely functions as a critical tethering factor, *via* its PHD and Tudor domains, to regulate DNA and histone methylation signals in breast cancer [33]. ASH1L, a member of the trithorax group, is the H3K36 methyltransferase. A recent study demonstrated that ASH1L is a crucial regulator of key leukemia target genes and contributes to leukemia pathogenesis [51]. Thus, these four PHFs are most likely proto-oncogenes in breast cancer.

In breast cancer, the two most commonly deleted/underexpressed PHF genes were *PHF23* and *CHD3*. Fusion of the *PHF23* gene with the *NUP98* (*Nucleoporin 98*) gene frequently recurs in acute myeloid leukemia [11]. PHF23 protein contains the canonical PHD finger at the C-terminus, and the PHD finger is retained in the *NUP98-PHF23* fusion. The leukemogenic potential of the NUP98-PHF23 fusion protein relies on the ability of the PHD finger to recognize the H3K4me3/2 marks [12]. CHD3, CHD4, and CHD5 proteins, which share common ATPase domains, tandem PHD domains, and chromodomains, are catalytic components of the NuRD (nucleosome remodeling histone deacetylase) complex [31]. CHD3 and CHD4 are expressed ubiquitously in every normal tissue, whereas CHD5 is preferentially expressed in the nervous system and testis. Both *CHD3* and *PHF23* genes are localized at 17p13.1, the TP53 region. A recent study demonstrated that deletions linked to TP53 loss drive cancer through p53-independent mechanisms [52]. In that study, a shRNA library targeting the ~100 protein-coding genes (excluding *TP53*) in mouse chromosome 11B3 syntenic to human 17p13.1 was screened for its tumor-suppressor activity in mouse models. Among 17 identified genes, CHD3 and PHF23 were considered potential tumor suppressors [52]. In the future, it will be important to determine how CHD3 and PHF23 play tumor-suppressor roles in a p53-dependent or independent manner in breast cancer.

In contrast to CHD3 and CHD5, which most likely function as tumor suppressors, the role of CHD4 in various tumors appears to be quite complex. It exhibits both oncogenic and tumor-suppressing properties [20, 31, 53-55]. Biochemically, the two PHD fingers of CHD4 are able to bind two distinct H3 tails with unmodified H3K4 and/or H3K9me3, but not H3K4me3 [56]. As the major component of NuRD, CHD4 has been implicated in regulating gene transcription and facilitating DNA repair [55, 56]. Compelling evidence indicates that CHD4 is a biomarker of drug sensitivity in cancer cells. For example, using high-throughput genomics, Geeleher *et al*. revealed that expression of CHD4 predicted the sensitivity of the histone deacetylase (HDAC) inhibitor Vorinostat in a large panel of cancer cell lines [57]. Furthermore, global proteomic analysis of the NCI-60 cancer cell lines revealed that the CHD4 protein is the second most recurrent and significant protein associated with the sensitivity of 97 different drugs [58]. Depletion of CHD4 sensitizes the CAMA1 breast cancer cell line to Vorinostat or leukemia cells to genotoxic agents and reduces tumor formation [53, 57]. In contrast to CHD3, the expression level of CHD4 was higher in Luminal, HER2+, and basal-like breast cancer compared with normal-like breast cancers (Supplementary Figure 10). Thus, CHD4 appears to play various roles in tumorigenesis and in the development of drug resistance.

In a recent study of 560 breast cancer samples with the whole-genome sequences, Nik-Zainal *et al.* identified 93 protein-coding cancers carrying probable driver mutations [59]. Five of them (*KMT2C*, *KMT2D*, *ASXL1*, *CREBBP*, and *PHF6*) were PHF genes. Our previous study, together with others, demonstrated that KMT2C and KMT2D function as tumor suppressors [29, 60]. The *ASXL1* is frequently mutated in a range of myeloid malignancies [21, 22]. Most cancer-associated ASXL1 mutations give rise to truncated proteins that retain the amino-terminal BAP1 (BRCA1 associated protein 1)-interacting region of ASXL1, but lose the carboxy-terminal PHD domain [21, 22, 61]. Accumulating data suggest that ASXL1 functions as a haploinsufficient tumor suppressor in the hematopoietic system [61]. In contrast, other studies suggested that *ASXL1* mutations might confer a gain-of-function, rather than loss-of-function, by generating a stable truncated protein lacking the PHD domain that either serves as a dominant negative function or generates a new function [61]. In this study, we also found that ASXL1 gene, localized at 20q11 region, is commonly amplified/gained in breast cancer (Table 1). Thus, functional roles of ASXL1 might be altered by gene amplification or gain-of-function mutations in a set of breast cancer.

A finding of particular interest from our study is the dysregulation of ZMYND8 in a subset of breast cancers. The ZMYND8 protein, which contains an N-terminal PHD-bromo-PWWP (PBP) cassette and a C-terminal

MYND (Myeloid-Nervy-DEAF1) domain (Supplementary Figure 11), has a wide range of interacting partners, including transcription factors, chromatin remodeling complexes, and histone demethylase/deacetylase enzymes. Analysis of the human endogenous coregulatory complexome revealed that the PBP cassette of ZMYND8 interacts directly with ERα, and both co-occupy a set of genomic sites of known ERα-binding sites [34]. Results of gene expression and reporter assay studies confirmed the coactivator functions of ZMYND8 for ER transcriptional activity [34]. A recent study revealed that ZMYND8 reads the dual histone modifications H3K4me1-H3K14ac via PHD-bromo cassette, and it is associated with repression of metastasis-linked genes [62]. The MYND domain of ZMYND8 is responsible for its interactions with the NuRD complex, notably the core catalytic component CHD4 [54]. Importantly, ZMYND8 has been identified as a new DDR (DNA Damage Response) factor; ZMYND8 recruits CHD4 to the sites of DNA damage and represses transcription and facilitates DNA repair by homologous recombination [54, 63]. Thus, ZMYND8 is implicated in both transcriptional activation and silencing, as well as DNA damage in a context-specific manner. In the present study, we provided additional evidence that ZMYND8 was involved in a positive feedback circuit involving the ER pathway in breast cancer, where the interaction between ZMYND8 and ERα promotes activation of the ERα target gene, including ZMYND itself. Furthermore, an additional regulatory mechanism, such as BRD4's association with super-enhancers, might regulate ZMYND expression in both ER-positive and ER-negative breast cancers. It is worth noting that, based on the NCBI database, at least 18 transcript variants of ZMYND8 exist. Western blot also revealed the additional protein bands with molecular weight lower than full-length ZMYND8 in breast cancer cell lines, particularly Luminal subtypes (Supplementary Figure 12). Furthermore, fusion of ZMYND8 has been identified in lymphoma and breast cancer; both of the ZMYND8 fusions reportedly remove the MYND domain but retain the N-terminal PBP cassette [64, 65]. ZMYND8 gene is localized at chromosomal region 20q13, which also contains candidate breast cancer oncogenes: *MYBL2* (*MYB proto-oncogene like 2*), *ZNF217* (*zinc finger protein 217*), and *AURKA* (aurora kinase A) [66]. As shown in Supplementary Figure 13, we found that most ZMYND8-amplified breast cancer samples also showed the amplification of other candidate oncogenes at the 20q13 region. It is necessary to further investigate whether and how ZMYND8 contribute, independently or cooperatively with other 20q13-amplified genes, to breast tumorigenesis.

In conclusion, our integrated genomic and transcriptomic analysis identified a broad spectrum of genetic alterations in PHF genes involved in different subtypes of breast cancer. Given the higher prevalence of genomic and transcriptomic alteration of several PHFs, such as PYGO2, ZMYND8, ASXL1, and CHD3, it is necessary to further characterize their structures, roles, and the molecular mechanisms of the PHD domain that are implicated in breast cancer initiation and progression. Furthermore, inhibition of critical epigenetic reader domains, such as PHD fingers, is emerging as the next frontier in epigenetic drug development. A few inhibitors that target PHD fingers, e.g., PYGO2-PHD and the third PHD of KDM5A, have been reported recently [67, 68]. We anticipate that our findings, along with similar studies on other types of tumors, will help prioritize which PHFs to study in order to better understand the molecular mechanisms and discover novel therapeutic targets in oncology.

MATERIALS AND METHODS

Cell culture

The cultures for the SUM series of breast cancer cell lines and the nontransformed human mammary epithelial cell line MCF10A were described previously [69]. The Colo824 cell line was obtained from DSMZ (Braunschweig, Germany), SUM cell lines were obtained from Dr. Stephen P. Ethier, and all other cell lines in this study were obtained from ATCC (Manassas, VA, USA). Cell growth was assessed by using an MTT assay. For clonogenic survival assays, cells were seeded in 12-well dishes and treated with JQ-1. After 12 days for SUM159 cells or 20 days for T47D cells, colonies were fixed and stained with 0.5% crystal violet. Cells were photographed and counted with an automated mammalian cell colony counter (Oxford Optronix GELCOUNT, Oxford, United Kingdom).

The cancer genome atlas (TCGA) data for breast cancer

The DNA copy number, mutation, and overall survival datasets of 960 breast cancer samples used in this research were obtained from the cBio Cancer Genomics Portal [26, 27]. For each PHF, the copy number was generated from the copy number analysis algorithm GISTIC (Genomic Identification of Significant Targets in Cancer) and categorized as copy number level per gene: "-2" is a deep loss (possibly a homozygous deletion), "-1" is a heterozygous deletion, "0" is diploid, "1" indicates a low-level gain, and "2" is a high-level amplification. For mRNA expression data, the relative expression of an individual gene and the distribution of a gene's expression in a reference population were analyzed. The reference population was either all tumors that are diploid for the gene in question or, when available, normal adjacent tissue. The returned value indicates the number of standard deviations away from the mean of expression in the reference population (Z-score). Somatic mutation data were obtained from exome sequencing [26, 27].

Breast cancer subtype and clinicopathologic information were extracted *via* the UCSC Cancer Genomics Browser (genome-cancer.ucsc.edu) and the cBio Cancer Genomics Portal [6, 26, 27]. Among the 960 breast cancer samples, 808 had subtype data available, including 22 normal-like, 405 Luminal A, 185 Luminal B, 66 HER2+, and 130 basal-like breast cancers [26, 33, 70].

The METABRIC (molecular taxonomy of breast cancer international consortium) dataset

The METABRIC dataset contains approximately 2000 primary breast cancers with long-term clinical follow-up data. METABRIC normal breast expression dataset (n = 144) was used as a non-cancer, tissue control. A detailed description of the dataset is presented in the original publication [28]. The CNAs and normalized expression data of METABRIC were downloaded with access permissions from the European Genome-phenome Archive (https://www.ebi.ac.uk/ega) under accession number EGAC00000000005. In the METABRIC dataset, copy number log_2 ratios were segmented using two analytical methods, circular binary segmentation and an adapted hidden Markov model. The median of the log_2 ratio was computed, and gene-centric alterations were categorized as amplification, gain, heterozygous loss, and homozygous loss. The data for 98 PHFs were based on the circular binary segmentation–derived copy number profiles [28]. The normalized gene expression profiles were generated using the Illumina Human HT-12 platform [28].

Semiquantitative PCR reactions

mRNA was prepared from human breast cancer cell lines and the MCF10A cell line by using an RNeasy Plus Mini Kit (QIAGEN). mRNA was mixed with qScript cDNA SuperMix (Quanta Biosciences, Gaithersburg, MD, USA) then converted into cDNA through a reverse-transcription (RT) reaction for real-time PCR reactions. Primer sets were ordered from Life Technologies (Carlsbad, CA, USA). A PUM1 primer set was used as a control. Semiquantitative RT-PCR was performed using the FastStart Universal SYBR Green Master (Roche Diagnostics, Indianapolis, IN, USA).

Immunoblotting and antibodies

Whole-cell lysates were prepared by scraping cells from dishes into cold RIPA lysis buffer. After centrifugation at high speed, protein content was estimated by the Bradford method. A total of 20–50 µg of total cell lysate was resolved by sodium dodecyl sulfate–polyacrylamide gel electrophoresis and transferred onto a polyvinylidene difluoride membrane. Antibodies used in the study included anti-ZMYND8 (1:1000, Bethyl Laboratories A302-089A, Montgomery, TX, USA), anti-MYC (1:1000, Cell Signaling D3N8F, Danvers, MA, USA), anti-ERα (1:1000, Cell Signaling 8644, Danvers, MA), anti-β-actin (1:5000, Sigma-Aldrich A5441, St. Louis, MO), and anti-β-tubulin (1:5000, Sigma-Aldrich T8328, St. Louis, MO, USA).

Statistical analysis

Statistical analyses were performed using R software (http://www.r-project.org) and Graphpad Prism (version 6.03). Correlations between copy numbers and mRNA levels of each PHF from TCGA breast cancer specimens were analyzed using Spearman, Kendall, and Pearson correlation tests. The Spearman and Kendall tests are rank correlations—the Spearman coefficient relates the two variables while conserving the order of data points, and the Kendall coefficient measures the number of ranks that match in the data set. We used the “cor” function in R statistical software for computation, specifying in the code which type of test we wanted (Spearman, Kendall, or Pearson). The significance of difference in mRNA expression level for each PHF among different subtypes and stages of breast cancer samples was calculated using ANOVA and Welch’s *t*-test. To analyze the relationships between PHF mRNA expression and overall patient survival in breast cancer, samples were divided into higher and lower expression groups of each PHF, based on mRNA expression Z-scores [RNA-Seq V2 RSEM (RNA-Seq by Expectation-Maximization)] in TCGA dataset or the log_2 normalized expression level in the METABRIC dataset. Multivariate survival analysis was conducted in TCGA breast cancer samples using the Cox regression function (“coxph”) in R. Factors included in the multivariate analysis model were age at diagnosis (continuous variable), ER status (positive vs. negative), progesterone receptor status (positive vs. negative), HER2 status (positive vs. negative), tumor size (>20 mm vs. ≤20 mm), lymph node status (positive vs. negative), metastasis status (positive vs. negative), and PAM50 subtype (basal vs. non-basal).

ACKNOWLEDGMENTS

This work was partially supported by grants from the NIH/NCI R21CA175244-01A1 and the Office of Research on Women’s Health (OWRH), the Department of Defense (DoD) Prostate Cancer Program PC121737 and PC130259 to Dr. Zeng-Quan Yang, and by grants from the NIH R01HL128647-01 to Dr. Zhe Yang, by funding from the China International Postdoctoral Exchange Fellowship Program to Huimei Yu, and the China Scholarship Council Program fellowship to Xiaofang Chu. We thank Hui Liu, Qin Ye, and Lihong Zhang for technical contributions. We thank Dr. Stephen P. Ethier for providing the SUM breast cancer cell lines.

CONFLICTS OF INTEREST

The authors declare no conflicts of interest.

REFERENCES

1. Greer EL, Shi Y. Histone methylation: a dynamic mark in health, disease and inheritance. Nat Rev Genet. 2012; 13:343-57. doi: 10.1038/nrg3173.
2. Feinberg AP, Koldobskiy MA, Gondor A. Epigenetic modulators, modifiers and mediators in cancer aetiology and progression. Nat Rev Genet. 2016; 17:284-99. doi: 10.1038/nrg.2016.13.
3. Musselman CA, Lalonde ME, Cote J, Kutateladze TG. Perceiving the epigenetic landscape through histone readers. Nat Struct Mol Biol. 2012; 19:1218-27. doi: 10.1038/nsmb.2436.
4. Patel DJ. A Structural Perspective on Readout of Epigenetic Histone and DNA Methylation Marks. Cold Spring Harb Perspect Biol. 2016; 8:a018754. doi: 10.1101/cshperspect.a018754.
5. Sanchez R, Zhou MM. The PHD finger: a versatile epigenome reader. Trends Biochem Sci. 2011; 36:364-72. doi: 10.1016/j.tibs.2011.03.005.
6. Cancer Genome Atlas N. Comprehensive molecular portraits of human breast tumours. Nature. 2012; 490:61-70. doi: 10.1038/nature11412.
7. Perou CM, Sorlie T, Eisen MB, van de Rijn M, Jeffrey SS, Rees CA, Pollack JR, Ross DT, Johnsen H, Akslen LA, Fluge O, Pergamenschikov A, Williams C, et al. Molecular portraits of human breast tumours. Nature. 2000; 406:747-52. doi: 10.1038/35021093.
8. Thomas C, Gustafsson JA. Estrogen receptor mutations and functional consequences for breast cancer. Trends Endocrinol Metab. 2015; 26:467-76. doi: 10.1016/j.tem.2015.06.007.
9. Ades F, Zardavas D, Bozovic-Spasojevic I, Pugliano L, Fumagalli D, de Azambuja E, Viale G, Sotiriou C, Piccart M. Luminal B breast cancer: molecular characterization, clinical management, and future perspectives. J Clin Oncol. 2014; 32:2794-803. doi: 10.1200/JCO.2013.54.1870.
10. Bertucci F, Finetti P, Birnbaum D. Basal breast cancer: a complex and deadly molecular subtype. Curr Mol Med. 2012; 12:96-110.
11. Ho H, Skaist AM, Pallavajjala A, Yonescu R, Batista D, Wheelan SJ, Ning Y. NUP98-PHF23 fusion is recurrent in acute myeloid leukemia and shares gene expression signature of leukemic stem cells. Leuk Res. 2016; 45:1-7. doi: 10.1016/j.leukres.2016.03.006.
12. Wang GG, Song J, Wang Z, Dormann HL, Casadio F, Li H, Luo JL, Patel DJ, Allis CD. Haematopoietic malignancies caused by dysregulation of a chromatin-binding PHD finger. Nature. 2009; 459:847-51. doi: 10.1038/nature08036.
13. Liu G, Bollig-Fischer A, Kreike B, van de Vijver MJ, Abrams J, Ethier SP, Yang ZQ. Genomic amplification and oncogenic properties of the GASC1 histone demethylase gene in breast cancer. Oncogene. 2009; 28:4491-500. doi: 10.1038/onc.2009.297.
14. Hou J, Wu J, Dombkowski A, Zhang K, Holowatyj A, Boerner JL, Yang ZQ. Genomic amplification and a role in drug-resistance for the KDM5A histone demethylase in breast cancer. Am J Transl Res. 2012; 4:247-56.
15. Huang Z, Wu H, Chuai S, Xu F, Yan F, Englund N, Wang Z, Zhang H, Fang M, Wang Y, Gu J, Zhang M, Yang T, et al. NSD2 is recruited through its PHD domain to oncogenic gene loci to drive multiple myeloma. Cancer Res. 2013; 73:6277-88. doi: 10.1158/0008-5472.CAN-13-1000.
16. Yang ZQ, Liu G, Bollig-Fischer A, Giroux CN, Ethier SP. Transforming properties of 8p11-12 amplified genes in human breast cancer. Cancer Res. 2010; 70:8487-97. doi: 10.1158/0008-5472.CAN-10-1013.
17. Mahmood SF, Gruel N, Nicolle R, Chapeaublanc E, Delattre O, Radvanyi F, Bernard-Pierrot I. PPAPDC1B and WHSC1L1 are common drivers of the 8p11-12 amplicon, not only in breast tumors but also in pancreatic adenocarcinomas and lung tumors. Am J Pathol. 2013; 183:1634-44. doi: 10.1016/j.ajpath.2013.07.028.
18. Richart L, Carrillo-de Santa Pau E, Rio-Machin A, de Andres MP, Cigudosa JC, Lobo VJ, Real FX. BPTF is required for c-MYC transcriptional activity and *in vivo* tumorigenesis. Nat Commun. 2016; 7:10153. doi: 10.1038/ncomms10153.
19. Paul S, Kuo A, Schalch T, Vogel H, Joshua-Tor L, McCombie WR, Gozani O, Hammell M, Mills AA. Chd5 requires PHD-mediated histone 3 binding for tumor suppression. Cell Rep. 2013; 3:92-102. doi: 10.1016/j.celrep.2012.12.009.
20. Kolla V, Zhuang T, Higashi M, Naraparaju K, Brodeur GM. Role of CHD5 in human cancers: 10 years later. Cancer Res. 2014; 74:652-8. doi: 10.1158/0008-5472.CAN-13-3056.
21. Gelsi-Boyer V, Brecqueville M, Devillier R, Murati A, Mozziconacci MJ, Birnbaum D. Mutations in ASXL1 are associated with poor prognosis across the spectrum of malignant myeloid diseases. J Hematol Oncol. 2012; 5:12. doi: 10.1186/1756-8722-5-12.
22. Gelsi-Boyer V, Trouplin V, Adelaide J, Bonansea J, Cervera N, Carbuccia N, Lagarde A, Prebet T, Nezri M, Sainty D, Olschwang S, Xerri L, Chaffanet M, et al. Mutations of polycomb-associated gene ASXL1 in myelodysplastic syndromes and chronic myelomonocytic leukaemia. Br J Haematol. 2009; 145:788-800. doi: 10.1111/j.1365-2141.2009.07697.x.
23. Beroukhim R, Mermel CH, Porter D, Wei G, Raychaudhuri S, Donovan J, Barretina J, Boehm JS, Dobson J, Urashima M, Mc Henry KT, Pinchback RM, Ligon AH, et al. The landscape of somatic copy-number alteration across human cancers. Nature. 2010; 463:899-905. doi: 10.1038/nature08822.

24. Ciriello G, Miller ML, Aksoy BA, Senbabaoglu Y, Schultz N, Sander C. Emerging landscape of oncogenic signatures across human cancers. Nat Genet. 2013; 45:1127-33. doi: 10.1038/ng.2762.

25. Liu L, Zhen XT, Denton E, Marsden BD, Schapira M. ChromoHub: a data hub for navigators of chromatin-mediated signalling. Bioinformatics. 2012; 28:2205-6. doi: 10.1093/bioinformatics/bts340.

26. Gao JJ, Aksoy BA, Dogrusoz U, Dresdner G, Gross B, Sumer SO, Sun YC, Jacobsen A, Sinha R, Larsson E, Cerami E, Sander C, Schultz N. Integrative Analysis of Complex Cancer Genomics and Clinical Profiles Using the cBioPortal. Science Signaling. 2013; 6.

27. Cerami E, Gao J, Dogrusoz U, Gross BE, Sumer SO, Aksoy BA, Jacobsen A, Byrne CJ, Heuer ML, Larsson E, Antipin Y, Reva B, Goldberg AP, et al. The cBio cancer genomics portal: an open platform for exploring multidimensional cancer genomics data. Cancer Discov. 2012; 2:401-4. doi: 10.1158/2159-8290.CD-12-0095.

28. Curtis C, Shah SP, Chin SF, Turashvili G, Rueda OM, Dunning MJ, Speed D, Lynch AG, Samarajiwa S, Yuan Y, Graf S, Ha G, Haffari G, et al. The genomic and transcriptomic architecture of 2,000 breast tumours reveals novel subgroups. Nature. 2012; 486:346-52. doi: 10.1038/nature10983.

29. Liu L, Kimball S, Liu H, Holowatyj A, Yang ZQ. Genetic alterations of histone lysine methyltransferases and their significance in breast cancer. Oncotarget. 2015; 6:2466-82. doi: 10.18632/oncotarget.2967.

30. Katoh M. Functional proteomics of the epigenetic regulators ASXL1, ASXL2 and ASXL3: a convergence of proteomics and epigenetics for translational medicine. Expert Rev Proteomics. 2015; 12:317-28. doi: 10.1586/14789450.2015.1033409.

31. Stanley FK, Moore S, Goodarzi AA. CHD chromatin remodelling enzymes and the DNA damage response. Mutat Res. 2013; 750:31-44. doi: 10.1016/j.mrfmmm.2013.07.008.

32. Li W, Mills AA. Architects of the genome: CHD dysfunction in cancer, developmental disorders and neurological syndromes. Epigenomics. 2014; 6:381-95. doi: 10.2217/epi.14.31.

33. Jiang Y, Liu L, Shan W, Yang ZQ. An integrated genomic analysis of Tudor domain-containing proteins identifies PHD finger protein 20-like 1 (PHF20L1) as a candidate oncogene in breast cancer. Mol Oncol. 2016; 10:292-302. doi: 10.1016/j.molonc.2015.10.013.

34. Malovannaya A, Lanz RB, Jung SY, Bulynko Y, Le NT, Chan DW, Ding C, Shi Y, Yucer N, Krenciute G, Kim BJ, Li C, Chen R, et al. Analysis of the human endogenous coregulator complexome. Cell. 2011; 145:787-99. doi: 10.1016/j.cell.2011.05.006.

35. Mohammed H, Russell IA, Stark R, Rueda OM, Hickey TE, Tarulli GA, Serandour AA, Birrell SN, Bruna A, Saadi A, Menon S, Hadfield J, Pugh M, et al. Progesterone receptor modulates ERalpha action in breast cancer. Nature. 2015; 523:313-7. doi: 10.1038/nature14583.

36. Ross-Innes CS, Stark R, Teschendorff AE, Holmes KA, Ali HR, Dunning MJ, Brown GD, Gojis O, Ellis IO, Green AR, Ali S, Chin SF, Palmieri C, et al. Differential oestrogen receptor binding is associated with clinical outcome in breast cancer. Nature. 2012; 481:389-93. doi: 10.1038/nature10730.

37. Gertz J, Savic D, Varley KE, Partridge EC, Safi A, Jain P, Cooper GM, Reddy TE, Crawford GE, Myers RM. Distinct properties of cell-type-specific and shared transcription factor binding sites. Mol Cell. 2013; 52:25-36. doi: 10.1016/j.molcel.2013.08.037.

38. Nagarajan S, Hossan T, Alawi M, Najafova Z, Indenbirken D, Bedi U, Taipaleenmaki H, Ben-Batalla I, Scheller M, Loges S, Knapp S, Hesse E, Chiang CM, et al. Bromodomain protein BRD4 is required for estrogen receptor-dependent enhancer activation and gene transcription. Cell Rep. 2014; 8:460-9. doi: 10.1016/j.celrep.2014.06.016.

39. Sanchez R, Meslamani J, Zhou MM. The bromodomain: from epigenome reader to druggable target. Biochim Biophys Acta. 2014; 1839:676-85. doi: 10.1016/j.bbagrm.2014.03.011.

40. Ali M, Hom RA, Blakeslee W, Ikenouye L, Kutateladze TG. Diverse functions of PHD fingers of the MLL/KMT2 subfamily. Biochim Biophys Acta. 2014; 1843:366-71. doi: 10.1016/j.bbamcr.2013.11.016.

41. Ruthenburg AJ, Li H, Milne TA, Dewell S, McGinty RK, Yuen M, Ueberheide B, Dou Y, Muir TW, Patel DJ, Allis CD. Recognition of a mononucleosomal histone modification pattern by BPTF via multivalent interactions. Cell. 2011; 145:692-706. doi: 10.1016/j.cell.2011.03.053.

42. Sun P, Watanabe K, Fallahi M, Lee B, Afetian ME, Rheaume C, Wu D, Horsley V, Dai X. Pygo2 regulates beta-catenin-induced activation of hair follicle stem/progenitor cells and skin hyperplasia. Proc Natl Acad Sci U S A. 2014; 111:10215-20. doi: 10.1073/pnas.1311395111.

43. Jessen S, Gu B, Dai X. Pygopus and the Wnt signaling pathway: a diverse set of connections. Bioessays. 2008; 30:448-56. doi: 10.1002/bies.20757.

44. Gu B, Sun P, Yuan Y, Moraes RC, Li A, Teng A, Agrawal A, Rheaume C, Bilanchone V, Veltmaat JM, Takemaru K, Millar S, Lee EY, et al. Pygo2 expands mammary progenitor cells by facilitating histone H3 K4 methylation. J Cell Biol. 2009; 185:811-26. doi: 10.1083/jcb.200810133.

45. Gu B, Watanabe K, Sun P, Fallahi M, Dai X. Chromatin effector Pygo2 mediates Wnt-notch crosstalk to suppress luminal/alveolar potential of mammary stem and basal cells. Cell Stem Cell. 2013; 13:48-61. doi: 10.1016/j.stem.2013.04.012.

46. Watanabe K, Fallahi M, Dai X. Chromatin effector Pygo2 regulates mammary tumor initiation and heterogeneity in MMTV-Wnt1 mice. Oncogene. 2014; 33:632-42. doi: 10.1038/onc.2012.620.

47. Lu PJ, Sundquist K, Baeckstrom D, Poulsom R, Hanby A, Meier-Ewert S, Jones T, Mitchell M, Pitha-Rowe P, Freemont P, Taylor-Papadimitriou J. A novel gene (PLU-1) containing highly conserved putative DNA chromatin binding motifs is specifically up-regulated in breast cancer.

Journal of Biological Chemistry. 1999; 274:15633-45. doi: DOI 10.1074/jbc.274.22.15633.

48. Yamane K, Tateishi K, Klose RJ, Fang J, Fabrizio LA, Erdjument-Bromage H, Taylor-Papadimitriou J, Tempst P, Zhang Y. PLU-1 is an H3K4 demethylase involved in transcriptional repression and breast cancer cell proliferation. Mol Cell. 2007; 25:801-12. doi: 10.1016/j.molcel.2007.03.001.

49. Yamamoto S, Wu Z, Russnes HG, Takagi S, Peluffo G, Vaske C, Zhao X, Moen Vollan HK, Maruyama R, Ekram MB, Sun H, Kim JH, Carver K, et al. JARID1B is a luminal lineage-driving oncogene in breast cancer. Cancer Cell. 2014; 25:762-77. doi: 10.1016/j.ccr.2014.04.024.

50. Koh JL, Brown KR, Sayad A, Kasimer D, Ketela T, Moffat J. COLT-Cancer: functional genetic screening resource for essential genes in human cancer cell lines. Nucleic Acids Res. 2012; 40:D957-63. doi: 10.1093/nar/gkr959.

51. Zhu L, Li Q, Wong SH, Huang M, Klein BJ, Shen J, Ikenouye L, Onishi M, Schneidawind D, Buechele C, Hansen L, Duque-Afonso J, Zhu F, et al. ASH1L Links Histone H3 Lysine 36 Dimethylation to MLL Leukemia. Cancer Discov. 2016; 6:770-83. doi: 10.1158/2159-8290.CD-16-0058.

52. Liu Y, Chen C, Xu Z, Scuoppo C, Rillahan CD, Gao J, Spitzer B, Bosbach B, Kastenhuber ER, Baslan T, Ackermann S, Cheng L, Wang Q, et al. Deletions linked to TP53 loss drive cancer through p53-independent mechanisms. Nature. 2016; 531:471-5. doi: 10.1038/nature17157.

53. Sperlazza J, Rahmani M, Beckta J, Aust M, Hawkins E, Wang SZ, Zu Zhu S, Podder S, Dumur C, Archer K, Grant S, Ginder GD. Depletion of the chromatin remodeler CHD4 sensitizes AML blasts to genotoxic agents and reduces tumor formation. Blood. 2015; 126:1462-72. doi: 10.1182/blood-2015-03-631606.

54. Gong F, Chiu LY, Cox B, Aymard F, Clouaire T, Leung JW, Cammarata M, Perez M, Agarwal P, Brodbelt JS, Legube G, Miller KM. Screen identifies bromodomain protein ZMYND8 in chromatin recognition of transcription-associated DNA damage that promotes homologous recombination. Genes Dev. 2015; 29:197-211. doi: 10.1101/gad.252189.114.

55. O'Shaughnessy A, Hendrich B. CHD4 in the DNA-damage response and cell cycle progression: not so NuRDy now. Biochem Soc Trans. 2013; 41:777-82. doi: 10.1042/BST20130027.

56. Torchy MP, Hamiche A, Klaholz BP. Structure and function insights into the NuRD chromatin remodeling complex. Cell Mol Life Sci. 2015; 72:2491-507. doi: 10.1007/s00018-015-1880-8.

57. Geeleher P, Lenkala D, Wang F, LaCroix B, Wang J, Karovic S, Maitland ML, Huang R, Loboda A, Nebozhyn M, Chisamore M, Hardwick J. Predicting Response to Histone Deacetylase Inhibitors Using High-Throughput Genomics. Journal of Investigative Medicine. 2015; 63: 683-4.

58. Gholami AM, Hahne H, Wu Z, Auer FJ, Meng C, Wilhelm M, Kuster B. Global proteome analysis of the NCI-60 cell line panel. Cell Rep. 2013; 4:609-20. doi: 10.1016/j.celrep.2013.07.018.

59. Nik-Zainal S, Davies H, Staaf J, Ramakrishna M, Glodzik D, Zou X, Martincorena I, Alexandrov LB, Martin S, Wedge DC, Van Loo P, Ju YS, Smid M, et al. Landscape of somatic mutations in 560 breast cancer whole-genome sequences. Nature. 2016; 534:47-54. doi: 10.1038/nature17676.

60. Ford DJ, Dingwall AK. The cancer COMPASS: navigating the functions of MLL complexes in cancer. Cancer Genet. 2015; 208:178-91. doi: 10.1016/j.cancergen.2015.01.005.

61. Micol JB, Abdel-Wahab O. The Role of Additional Sex Combs-Like Proteins in Cancer. Cold Spring Harb Perspect Med. 2016; 6. doi: 10.1101/cshperspect.a026526.

62. Li N, Li Y, Lv J, Zheng X, Wen H, Shen H, Zhu G, Chen TY, Dhar SS, Kan PY, Wang Z, Shiekhattar R, Shi X, et al. ZMYND8 Reads the Dual Histone Mark H3K4me1-H3K14ac to Antagonize the Expression of Metastasis-Linked Genes. Mol Cell. 2016; 63:470-84. doi: 10.1016/j.molcel.2016.06.035.

63. Spruijt CG, Luijsterburg MS, Menafra R, Lindeboom RG, Jansen PW, Edupuganti RR, Baltissen MP, Wiegant WW, Voelker-Albert MC, Matarese F, Mensinga A, Poser I, Vos HR, et al. ZMYND8 Co-localizes with NuRD on Target Genes and Regulates Poly(ADP-Ribose)-Dependent Recruitment of GATAD2A/NuRD to Sites of DNA Damage. Cell Rep. 2016; 17:783-98. doi: 10.1016/j.celrep.2016.09.037.

64. Panagopoulos I, Micci F, Thorsen J, Haugom L, Buechner J, Kerndrup G, Tierens A, Zeller B, Heim S. Fusion of ZMYND8 and RELA genes in acute erythroid leukemia. PLoS One. 2013; 8:e63663. doi: 10.1371/journal.pone.0063663.

65. Wada Y, Matsuura M, Sugawara M, Ushijima M, Miyata S, Nagasaki K, Noda T, Miki Y. Development of detection method for novel fusion gene using GeneChip exon array. J Clin Bioinforma. 2014; 4:3. doi: 10.1186/2043-9113-4-3.

66. Ginestier C, Cervera N, Finetti P, Esteyries S, Esterni B, Adelaide J, Xerri L, Viens P, Jacquemier J, Charafe-Jauffret E, Chaffanet M, Birnbaum D, Bertucci F. Prognosis and gene expression profiling of 20q13-amplified breast cancers. Clin Cancer Res. 2006; 12:4533-44. doi: 10.1158/1078-0432.CCR-05-2339.

67. Wagner EK, Nath N, Flemming R, Feltenberger JB, Denu JM. Identification and characterization of small molecule inhibitors of a plant homeodomain finger. Biochemistry. 2012; 51:8293-306. doi: 10.1021/bi3009278.

68. Miller TC, Rutherford TJ, Birchall K, Chugh J, Fiedler M, Bienz M. Competitive binding of a benzimidazole to the histone-binding pocket of the Pygo PHD finger. ACS Chem Biol. 2014; 9:2864-74. doi: 10.1021/cb500585s.

69. Yang ZQ, Streicher KL, Ray ME, Abrams J, Ethier SP. Multiple interacting oncogenes on the 8p11-p12 amplicon in human breast cancer. Cancer Res. 2006; 66:11632-43. doi: 10.1158/0008-5472.CAN-06-2946.

70. Liu H, Liu L, Holowatyj A, Jiang Y, Yang ZQ. Integrated genomic and functional analyses of histone demethylases identify oncogenic KDM2A isoform in breast cancer. Mol Carcinog. 2016; 55:977-90. doi: 10.1002/mc.22341.
